# RASSF8-mediated transport of Echinoid via the exocyst promotes *Drosophila* wing elongation and epithelial ordering

**DOI:** 10.1242/dev.199731

**Published:** 2021-10-14

**Authors:** Eunice H. Y. Chan, Yanxiang Zhou, Birgit L. Aerne, Maxine V. Holder, Anne Weston, David J. Barry, Lucy Collinson, Nicolas Tapon

**Affiliations:** 1 Apoptosis and Proliferation Control Laboratory, The Francis Crick Institute, 1 Midland Road, London NW1 1AT, UK; 2 Electron Microscopy Science Technology Platform, The Francis Crick Institute, 1 Midland Road, London NW1 1AT, UK; 3 Advanced Light Microscopy Science Technology Platform, The Francis Crick Institute, 1 Midland Road, London NW1 1AT, UK

**Keywords:** Development, *Drosophila*, Wing, Cell junctions, Epithelium, Exocyst

## Abstract

Cell-cell junctions are dynamic structures that maintain cell cohesion and shape in epithelial tissues. During development, junctions undergo extensive rearrangements to drive the epithelial remodelling required for morphogenesis. This is particularly evident during axis elongation, where neighbour exchanges, cell-cell rearrangements and oriented cell divisions lead to large-scale alterations in tissue shape. Polarised vesicle trafficking of junctional components by the exocyst complex has been proposed to promote junctional rearrangements during epithelial remodelling, but the receptors that allow exocyst docking to the target membranes remain poorly understood. Here, we show that the adherens junction component Ras Association domain family 8 (RASSF8) is required for the epithelial re-ordering that occurs during *Drosophila* pupal wing proximo-distal elongation. We identify the exocyst component Sec15 as a RASSF8 interactor. Loss of *RASSF8* elicits cytoplasmic accumulation of Sec15 and Rab11-containing vesicles. These vesicles also contain the nectin-like homophilic adhesion molecule Echinoid, the depletion of which phenocopies the wing elongation and epithelial packing defects observed in *RASSF8* mutants. Thus, our results suggest that RASSF8 promotes exocyst-dependent docking of Echinoid-containing vesicles during morphogenesis.

## Abstract (precis)

**Summary:** Cell junction remodelling is essential for tissues to acquire their shape during development. RASSF8 is required for exocyst-mediated transport of the adherens junction component Echinoid to promote *Drosophila* wing elongation.

## INTRODUCTION

The control of tissue shape during morphogenesis is one of the most complex questions in developmental biology. In epithelial tissues, cells adhere to each other through dynamic apical E-cadherin (Ecad)-containing adherens junctions (AJs) anchored to the underlying actin cytoskeleton (Charras and Yap, 2018; Rusu and Georgiou, 2020). Overall tissue shape is determined by polarised and coordinated cell behaviours, such as oriented cell divisions and cell-cell rearrangements (Paré and Zallen, 2020; Perez-Vale and Peifer, 2020; van Leen et al., 2020). These planar polarised behaviours are driven by differential modulation of local actomyosin and adhesion dynamics, as well as by large-scale tissue rearrangements. In *Drosophila* epithelia, the importance of polarised cell behaviours in tissue axis elongation has been demonstrated in several tissues, including the embryonic epidermis, notum and wing (Diaz de la Loza and Thompson, 2017; Mao and Lecuit, 2016; Paré and Zallen, 2020).

**Fig. 1.**
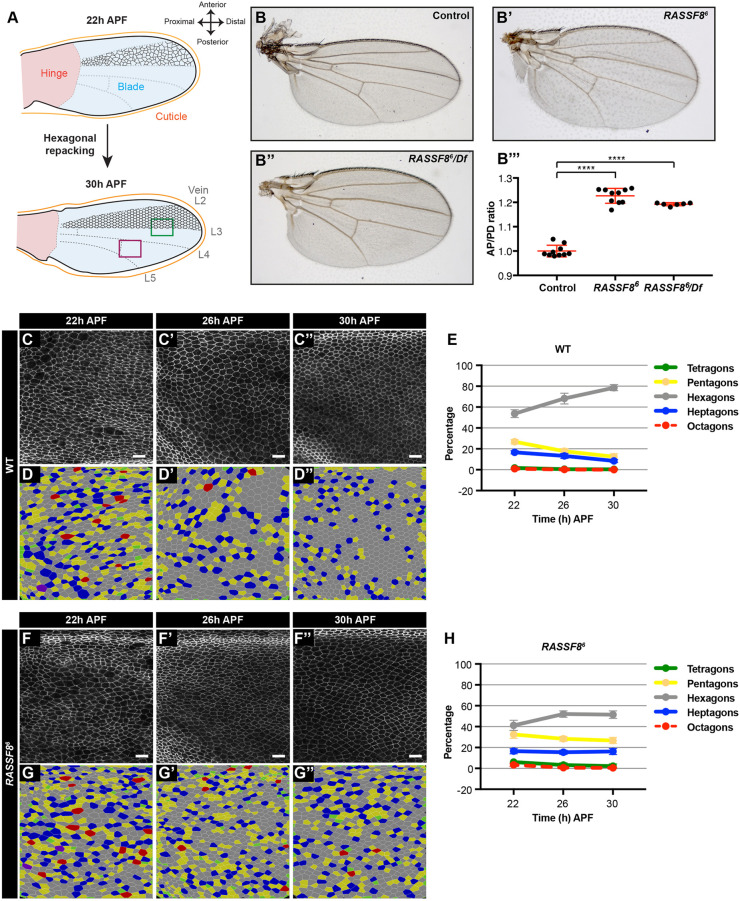
**RASSF8 is required for pupal wing cell hexagonal packing.** (A) Schematic diagram of pupal wing morphology, axes and development from 22 to 30 h after puparium formation (APF). Colour-coded rectangles indicate regions imaged for analyses. The region distal to the posterior crossvein is marked in purple and the region straddling the L3 vein is marked by a green rectangle. The positions of the longitudinal veins (L2-L5) and crossveins are indicated as dashed lines. As the wing hinge (shaded pink) contracts, the wing blade (shaded blue) extends in the PD axis and the wing epithelial cells (indicated in black between L2 and L3) reorder to form a hexagonal lattice. The wing images throughout this article are oriented as indicated in this diagram. (B) Wild-type wing, (B′) *RASSF8^6^* homozygous mutant wing and (B″) *RASSF8^6^*/*Df (3R)BSC321* deficiency heterozygous wing. (B‴) Quantification of relative wing roundness (ratio of AP to PD axis, normalised so that wild-type ratio=1). Data are mean±s.d. ANOVA (Tukey's correction): ****P<0.0001. As expected, because *RASSF8^6^* is a null mutant, the homozygous *RASSF8^6^* animals have a similar phenotype to the *RASSF8^6^*/Df animals. (C-F) Hexagonal cell packing of wild-type and *RASSF8* mutant wings at 22, 26 and 30 h APF. Images of Ecad::GFP-labelled wild-type (C-C″) and *RASSF8* mutant (F-F″) wings at a region distal to the posterior crossvein (purple rectangle in A). Colour-coded images indicate the number of neighbours for each cell in wild-type (D-D″) and *RASSF8* mutant (G-G″) wings, determined by using Tissue Analyzer (Aigouy et al., 2010). (E,H) Percentage of cells with four, five, six, seven and eight neighbours (colour coded as indicated) in wild type (D) and *RASSF8* mutants (G). The red line (octagons) is dashed so the green line (tetragons) can be seen. Data are mean±s.d., *n*=1500-5000 cells from three to five individual wings. Scale bars: 10 μm. See Table S1 for raw data.

The proximo-distal (PD) extension of the *Drosophila* pupal wing has emerged as a powerful system in which to study epithelial remodelling (Diaz de la Loza and Thompson, 2017; Eaton and Jülicher, 2011). The adult wing blade develops from a structure called the pouch in the wing imaginal disc. The wing disc is an epithelial sac in the larva that will give rise to the wing blade, the wing hinge (the connection between the blade and thorax) and part of the thorax (Fig. 1A, upper panel) (Held, 2002). During the pupal stages of development, the wing blade acquires its final elongated shape through the contraction of the hinge (Fig. 1A) (Aigouy et al., 2010). Hinge contraction results in pulling of the wing blade against the resistance of the distal wing tip, which is tethered to the chitinous pupal cuticle via the apical extracellular matrix component Dumpy (Diaz-de-la-Loza et al., 2018; Etournay et al., 2015; Ray et al., 2015). This elongation causes both oriented cell divisions along the PD axis and widespread cell-cell rearrangements throughout the wing, ultimately reordering the wing cells from a relatively disorganised array of polygons to a highly regular hexagonal lattice (Fig. 1A, lower panel) (Aigouy et al., 2010; Classen et al., 2005; Diaz-de-la-Loza et al., 2018; Etournay et al., 2016, 2015; Guirao et al., 2015; Ray et al., 2015).

Epithelial reordering during pupal wing elongation requires polarised actomyosin contractility and recycling of AJ components (Aigouy et al., 2010; Bardet et al., 2013; Classen et al., 2005; Ma et al., 2008; Warrington et al., 2013). The lipid phosphatase PTEN clears Rho kinase and Myosin II from elongating junctions following neighbour exchanges (T1 transitions), and its depletion causes a failure of epithelial reordering (Bardet et al., 2013). Recycling of AJ components is thought to depend on the Frizzled (Fz) ‘core’ and on Fat/Dachsous (Ds) planar cell polarity (PCP) signalling pathways (Aigouy et al., 2010; Classen et al., 2005; Gault et al., 2012; Ma et al., 2008; Warrington et al., 2013). The planar polarised seven-pass transmembrane protein Fz recruits Rho guanine nucleotide exchange factor 2 (RhoGEF2) via the scaffold protein Dishevelled (Dsh), which in turn promotes actomyosin-dependent Ecad endocytosis (Warrington et al., 2013). Flamingo (Fmi, also known as Starry night), another core PCP transmembrane protein, has been suggested to promote Ecad exocytosis in the pupal wing by recruiting the exocyst component Sec5 (Classen et al., 2005).

Polarised exocytosis is key to apico-basal polarity establishment and maintenance, as well as tissue remodelling (Polgar and Fogelgren, 2018; Román-Fernández and Bryant, 2016). The exocyst is an octameric protein complex first identified in yeast genetic screens for secretory mutants (TerBush et al., 1996; TerBush and Novick, 1995). The exocyst mediates docking of post-Golgi vesicles and Rab11-positive recycling endosomes to the plasma membrane and promotes SNARE (soluble NSF attachment protein receptor) fusion complex activation (Heider and Munson, 2012; Zeng et al., 2017). Rab11 and the exocyst have been implicated in targeting vesicles and their cargoes to a variety of subcellular locations in higher eukaryotes, including the basolateral (Grindstaff et al., 1998; Lipschutz et al., 2000) and junctional/apical domains (Ahmed and Macara, 2017; Blankenship et al., 2007; Bryant et al., 2010; Campbell et al., 2009; Classen et al., 2005; Langevin et al., 2005; Mateus et al., 2011; Oztan et al., 2007; Xiong et al., 2012; Yeaman et al., 2004) in epithelial cells; the base of cilia (Lipschutz, 2019); the leading edge of migrating fibroblasts (Zago et al., 2019); nascent axonal tips (Lalli, 2009; Mehta et al., 2005; Murthy et al., 2003, 2005); and photoreceptor rhabdomeres (Beronja et al., 2005; Satoh et al., 2005; Wu et al., 2005). Correctly delivering exocyst cargoes to these different locations is therefore crucial to maintain polarity and orderly developmental tissue remodelling.

Recognition of the correct target membranes is based both on interaction of the exocyst subunits Exo70 and Sec3 with phosphatidylinositol(4,5)-bisphosphate (He et al., 2007; Liu et al., 2007; Pleskot et al., 2015; Zhang et al., 2008), as well as binding of exocyst components to proteins localised at the target site. These proteins include small GTPases, such as Cdc42 in yeast and higher eukaryotes (Zeng et al., 2017), or polarity determinants, such as Par3 (Ahmed and Macara, 2017; Polgar and Fogelgren, 2018). However, for numerous exocyst target sites and cargoes, the nature of the docking cues is unknown.

We have previously identified the N-terminal RA (Ras association) domain-containing protein RASSF8 as an AJ component required for morphogenesis during *Drosophila* retinal development (Langton et al., 2009). *RASSF8* mutants display cell adhesion defects as indicated by broken AJs during retinal remodelling (Langton et al., 2009). RASSF8 physically interacts with two other AJ-localised scaffold proteins: ASPP and Magi (Langton et al., 2007; Zaessinger et al., 2015). This complex promotes Ecad stability at AJs by recruiting the Par3 ortholog Bazooka (Baz) (Zaessinger et al., 2015) and antagonising Src activity via C-terminal Src kinase (Csk) (Langton et al., 2007, 2009). Intriguingly, RASSF8 also has ASPP-independent functions, as *RASSF8* mutant flies, unlike *ASPP* mutants, have a broad wing phenotype, which is indicative of abnormal PD axis extension (Langton et al., 2009 and this study). Here, we explore the functions of RASSF8 in wing development. We find that RASSF8 physically interacts with the exocyst component Sec15 and is required for trafficking of junctional components through Rab11 vesicles. Loss of *RASSF8* results in cytoplasmic accumulation of the adhesion molecule Echinoid (Ed) in enlarged Rab11-positive compartments. Furthermore, *ed* depletion in the wing blade leads to similar hexagonal packing and PD axis extension defects to those observed in *RASSF8* mutants. Thus, RASSF8 and Sec15 function together in promoting the Rab11-mediated trafficking of Ed during wing morphogenesis, suggesting that RASSF8, like its binding partner Baz/Par3, can act as an AJ receptor for exocyst-dependent membrane trafficking.

## RESULTS

### *RASSF8* mutant wings have an abnormal aspect ratio and hexagonal packing defects

We previously reported that *RASSF8* mutant adult wings have both overgrowth and broad wing phenotypes (Langton et al., 2009). We quantified the shape defect by calculating the ratio between the antero-posterior (AP) and PD axes, and observed a 20% increase in AP to PD ratio in *RASSF8* mutants (Fig. 1B-B‴). PD axis elongation during pupal wing development involves epithelial reordering induced by hinge contraction to yield a highly organised hexagonal lattice (Aigouy et al., 2010; Classen et al., 2005). To test whether *RASSF8* mutants present defects in this process, we imaged the AJs of wild-type and *RASSF8* mutant pupal wings using an endogenously tagged *Ecad::GFP* knock-in line (Huang et al., 2009) and quantified the polygon distributions of the cell population between veins L4 and L5, distal to the posterior crossvein (Fig. 1A, purple rectangle) at 22, 26 and 30 h after puparium formation (APF) (Fig. 1C-H). The polygon distribution indicates the number of neighbours of each individual cell, from tetragons (four neighbours) to octagons (eight neighbours). As previously reported (Aigouy et al., 2010; Classen et al., 2005), the proportion of hexagonal cells increases with time in wild-type wings (Fig. 1C-D). At 30 h APF, about 80% of cells achieved hexagonal packing (Fig. 1E). In contrast, the polygon distribution in *RASSF8* mutants remains relatively stagnant, with around 50% of cells attaining a hexagonal shape at 30 h APF (Fig. 1F-H). We observed a similar defect in cell packing across the L3 vein of the wing (Fig. 1A, green rectangle, Fig. S1A-E′). Analysis of *RASSF8* mutant clones suggests that this hexagonal patterning defect is cell-autonomous, as the surrounding wild-type tissue is not affected (Fig. S1F-F″). Junctional Ecad intensity was not changed in *RASSF8* mutant pupal wing clones compared with control (Fig. S1F‴). Thus, RASSF8 is required for the maturation of the hexagonal lattice in the pupal wing.

The best characterised binding partner for RASSF8 is the scaffold protein ASPP, and both proteins function together during retinal morphogenesis (Langton et al., 2009). However, loss of *ASPP* results in a very mild hexagonal packing defect (∼70% hexagons at 30 h APF, Fig. S1G-K′), which may be due to the fact that junctional RASSF8 levels are reduced in ASPP mutant tissue (Langton et al., 2009). This suggests that RASSF8 acts independently of ASPP during wing morphogenesis.

### RASSF8 interacts with Sec15 independently of Rab11

**Fig. 2.**
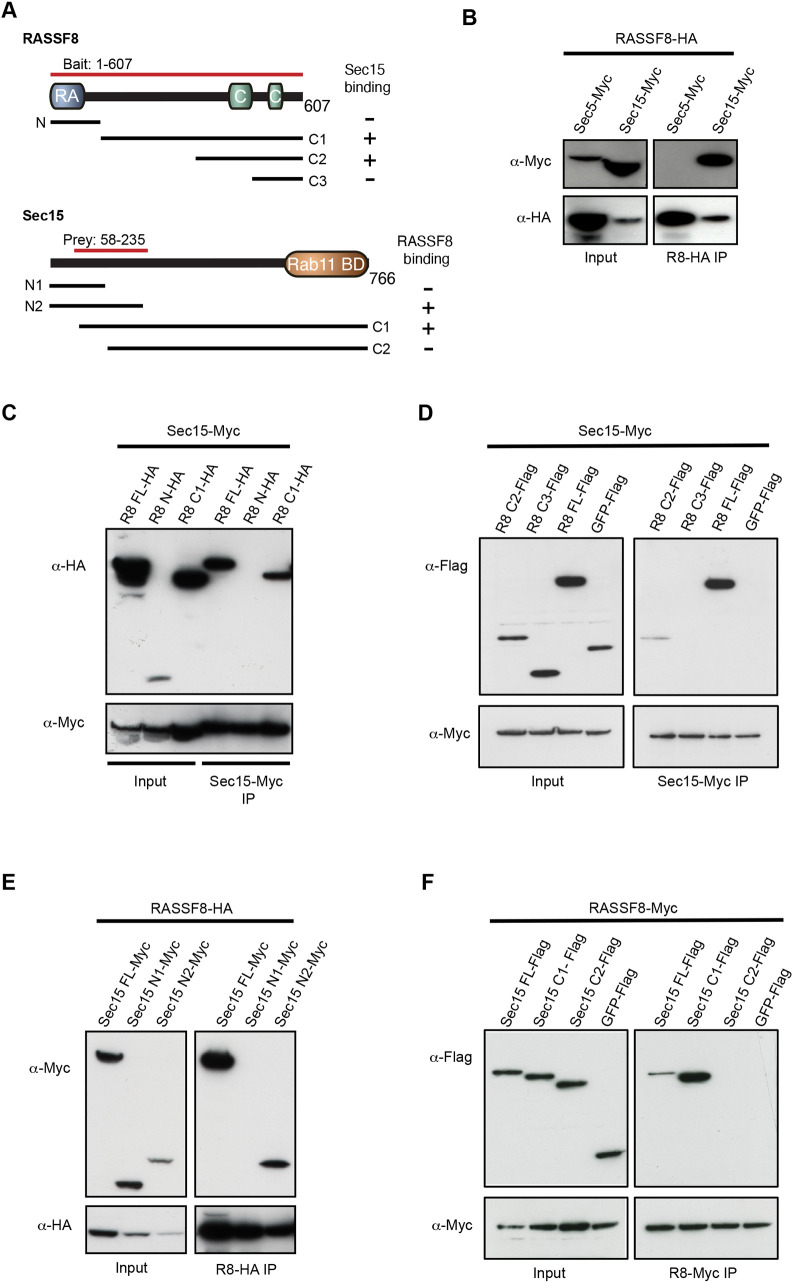
**Identification of Sec15 as a RASSF8 partner.** (A) Schematic diagram of the RASSF8 and Sec15 proteins. The two-hybrid bait and prey, and the constructs used in the co-immunoprecipitation experiments are also shown [RASSF8: N, (amino acids) 1-120; C1, 121-607; C2, 350-607; C3, 490-607; Sec15: N1, 1-134; N2, 1-225; C1, 58-766; C2, 130-766]. (B-F) Co-immunoprecipitation experiment in S2 cells overexpressing the indicated constructs. (B) RASSF8-HA binds specifically to Sec15-Myc but not to Sec5-Myc. (C,D) Amino acids 350-490 of RASSF8 are necessary for Sec15 binding. (E,F) Amino acids 135-225 of Sec15 are required for its interaction with RASSF8.

To explore the molecular mechanism by which RASSF8 controls hexagonal cell packing, we carried out a yeast-two hybrid screen using full-length *Drosophila* RASSF8 as a bait. In addition to ASPP, an established RASSF8 binding partner (Langton et al., 2009), we identified the exocyst subunit Sec15 (amino acids 59-234) as a RASSF8 interactor (Fig. 2A). To confirm this interaction, we co-expressed HA-tagged RASSF8 together with either Myc-tagged Sec15 or Sec5 in *Drosophila* S2 cells and performed co-immunoprecipitation (co-IP) experiments. We detected Sec15 but not Sec5 in the RASSF8 immunoprecipitates, confirming the RASSF8/Sec15 association (Fig. 2B). Sec15 binds the small GTPase Rab11 via its C-terminus, and this interaction is essential for polarised trafficking during sensory organ precursor (SOP) asymmetric division (Jafar-Nejad et al., 2005), in neurons (Mehta et al., 2005) and for AJ recycling of Ecad in the notum (Langevin et al., 2005). As Rab11 inactivation prevents junctional remodelling and hexagonal packing in the pupal wing (Classen et al., 2005), we decided to further characterise the RASSF8/Sec15 interaction.

To map the domains required for the interaction between Sec15 and RASSF8, we carried out co-IP experiments using fragments of either protein. These experiments show that RASSF8 amino acids 350-490 are required for binding to Sec15, while the RA domain is dispensable (Fig. 2C,D). Sec15 amino acids 58-225 mediate binding to RASSF8 (Fig. 2E,F), which is distinct from the Rab11 binding domain of Sec15 (amino acids 565-764) (Wu et al., 2005). As previous studies had shown that Sec15 binds specifically to the GTP-bound form of Rab11 (Langevin et al., 2005; Wu et al., 2005; Zhang et al., 2004) and RASSF8 contains a RA domain, which could potentially bind to Ras family GTPases (Ponting and Benjamin, 1996), we tested whether RASSF8 and Rab11 can directly associate. Although we detected a preferential binding of Sec15 to GTP-bound Rab11 (Fig. S2A), as previously described, no obvious interaction was detected between RASSF8 and Rab11 (GTP or GDP bound; Fig. S2B). Thus, our data suggest that RASSF8 interacts with the exocyst component Sec15, independently of Rab11.

### Rab11 and Sec15 accumulate in *RASSF8* clones

**Fig. 3.**
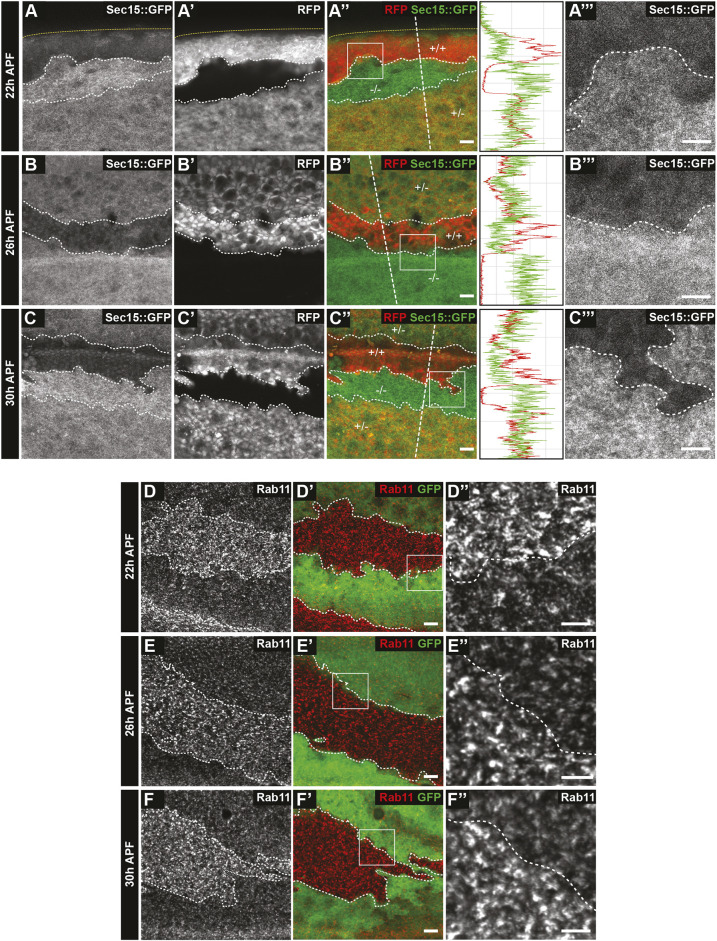
**Accumulation of Sec15 and Rab11 in *RASSF8* mutant pupal wing clones.** (A-C‴) Increase in Sec15::GFP (driven by the *ubiquitin* promoter) in *RASSF8* mutant clones (negative for RFP in red) at 22 (A-A″), 26 (B-B″) and 30 (C-C″) h APF. Clone boundaries are marked by white dotted lines. Yellow dotted lines at 22 h AFP show the edge of the wing. In the merge channel, the genotypes of the clones are given [+/+, wild type (two copies of RFP); +/−, heterozygous (one copy of RFP); −/−, homozygous *RASSF8* mutant (no copies of RFP)]. A‴, B‴ and C‴ are zoomed-in views of the boxed areas in A″, B″ and C″, respectively. Traces show the intensity profiles at the straight white dashed lines in the merged images using Fiji. (D-F″) Accumulation of Rab11 in *RASSF8* mutant clones. Rab11 antibody staining in *RASSF8* mutant clones marked by the absence of GFP at 22 (D-D″), 26 (E-E″) and 30 (F-F″) h APF. D″, E″ and F″ are zoomed-in views of the boxed areas in D′,E and F′, respectively. Scale bars: 10 μm in A,A″,B,B″,C,C″,D,D′,E,E′,F,F′; 7 μm in A‴,B‴,C‴,D″,E″,F″.

**Fig. 4.**
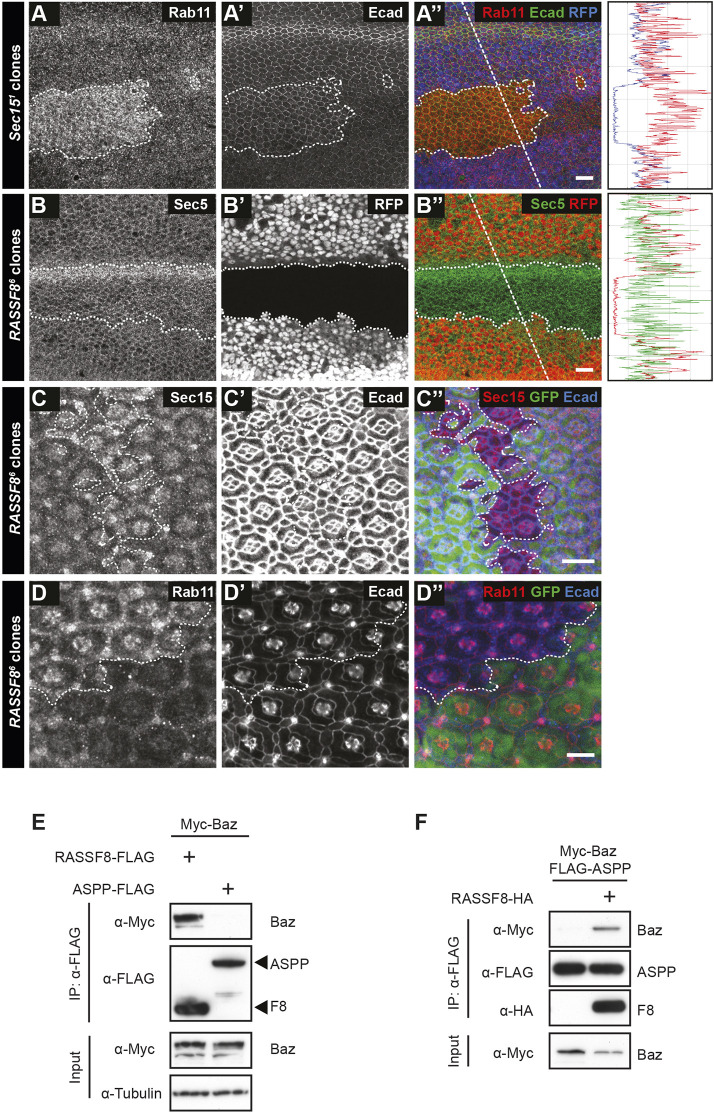
**Rab11 and Sec15, but not Sec5, are mislocalised in *RASSF8* mutant clones.** (A-D″) Confocal micrographs of pupal wing discs and pupal retinas at 26 h APF bearing *sec15* (A-A″) or *RASSF8* (B-D″) mutant clones generated using *hsFLP* (wing clones) or *eyFLP* (retinal clones) and stained as indicated. White dotted lines indicate the clone boundaries. (A-A″) Accumulation of Rab11 in *sec15* mutant clones marked by the absence of RFP in the pupal wing. (B-B″) Sec5 staining is not affected in *RASSF8* mutant pupal wing clones marked by the absence of RFP. The horizontal band of elevated Sec5 intensity in the mutant clones corresponds to a wing vein. In A-B″, the traces show the intensity profiles at the straight white dotted lines in the merged image using Fiji. (C-D″) Accumulation of Sec15 (C-C″) and Rab11 (D-D″) in *RASSF8* mutant clones marked by the absence of GFP in pupal retinas. Scale bars: 10 μm. (E,F) Co-immunoprecipitation experiments in S2 cells overexpressing the indicated constructs. (E) Baz associates with RASSF8 but not with ASPP. (F) ASPP co-immunoprecipitates Baz only in the presence of RASSF8.

As RASSF8 binds to Sec15, we tested whether the localisation of Rab11 or Sec15 is affected in *RASSF8* mutant clones. We observed cytoplasmic accumulation of Sec15::GFP (expressed under the *ubiquitin-63E* promoter – see Materials and Methods) and Rab11 in *RASSF8* clones at various time points (Fig. 3A-F″). In the case of Sec15, the heterozygous tissue already displayed a marked cytoplasmic accumulation, showing that Sec15 is extremely sensitive to RASSF8 dose and supporting the idea that these proteins physically interact. In agreement with what has been described in the pupal notum (Langevin et al., 2005), we observed an accumulation of intracellular Rab11 within Sec15 mutant clones in the pupal wing (Fig. 4A-A″). Together with the fact that dominant-negative Rab11 also prevents hexagonal packing (Classen et al., 2005), this suggests that RASSF8 is required for exocyst-dependent trafficking of Rab11 vesicles.

In budding yeast, some exocyst subunits (Sec3p and Exo70p) are at the exocytosis target site, whereas others are associated with the cargo vesicle, suggesting that full exocyst assembly occurs at the membrane upon vesicle docking (Boyd et al., 2004; Liu et al., 2018). Indeed, in *Drosophila*, Sec15 is primarily vesicular (Jafar-Nejad et al., 2005; Langevin et al., 2005), while several other subunits (Sec5, Sec6 and Sec8) are at least partly membrane associated (Beronja et al., 2005; Langevin et al., 2005; Murthy et al., 2005). Interestingly, the localisation of Sec5, which is primarily cortical in the pupal wing, is not altered in *RASSF8* mutant clones, suggesting that RASSF8 is required for the localisation of a subset of exocyst components (Fig. 4B,B′).

We have previously shown that *RASSF8* mutant clones have patterning defects in retinal development at the pupal stage (26-27 h APF) (Langton et al., 2009). In this system, we also observed Sec15 and Rab11 accumulation in *RASSF8* mutant clones, suggesting that the RASSF8 requirement for exocyst function is not confined to the wing (Fig. 4C-D″). In contrast, the markers of early endosomes (Rab5) and mature endosomes (Rab7 and Hrs) are not altered in *RASSF8* retinal clones, showing that the Sec15/Rab11 defect is not indicative of a general disruption in vesicle trafficking (Fig. S3A-C″). Consistent with a defect in cell-cell contacts, transmission electron microscopy (TEM) of pupal retinas revealed gaps between *RASSF8* mutant cells (Fig. S4A-E).

### RASSF8 is implicated in exocyst function independently of Bazooka/Par3

Together, our findings are consistent with a subset of Rab11 vesicles failing to be correctly targeted to the plasma membrane in *RASSF8* mutants. As RASSF8 is localised at the cell cortex (AJs) in the wing and eye (Langton et al., 2009), this suggests that RASSF8 may act as a cortical receptor for exocyst docking. Interestingly, the polarity protein Par3 has recently been shown to act as an exocyst receptor in mouse mammary epithelial cells by interacting directly with Exo70 (Ahmed and Macara, 2017). This warranted further investigation, as ASPP2, the mammalian homolog of the RASSF8 partner ASPP, has been reported to associate with Par3 (Cong et al., 2010; Sottocornola et al., 2010). In addition, we had reported that a complex comprising the scaffold protein Magi, ASPP and RASSF8 is required for the correct recruitment of the Par3 ortholog Baz to the AJs during retinal morphogenesis (Zaessinger et al., 2015). Finally, we have shown that the RASSF8 paralog Meru directly binds to Baz to induce its planar polarisation in *Drosophila* sensory organ precursor cells (Banerjee et al., 2017). This suggests that N-terminal RASSF proteins have a general function in Par3/Baz recruitment.

**Fig. 5.**
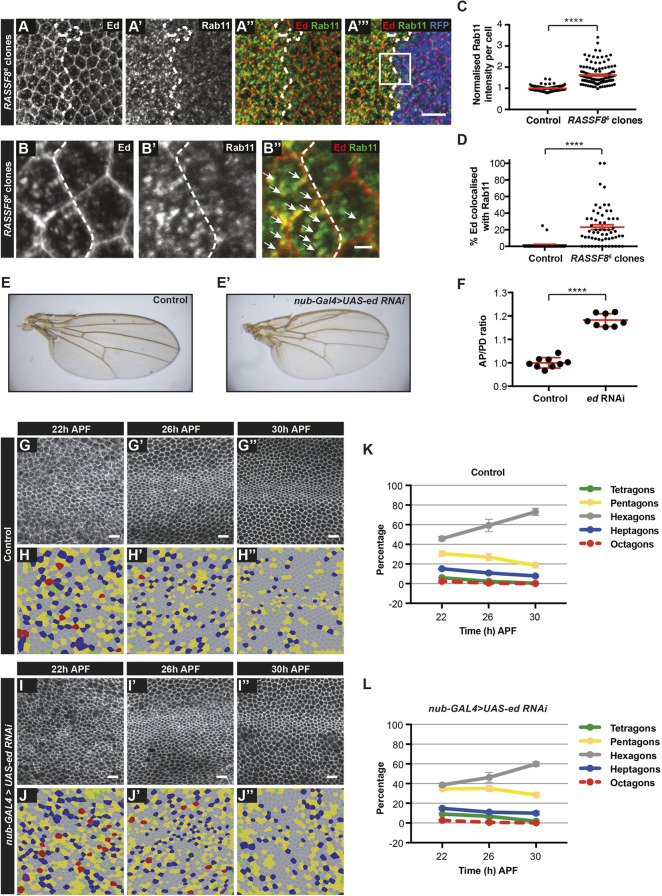
**Ed accumulates in Rab11 compartments in *RASSF8* mutants.** (A-A‴) Accumulation of Ed and Rab11 in *RASSF8* mutant clones. Ed (red) and Rab11 (green) antibody staining in *RASSF8* mutant clones (marked by absence of RFP in blue). Clone boundaries are marked by white dotted lines. (B-B″) Zoomed-in views of the images in A-A‴ (see box in A‴) with colocalisation of Ed and Rab11 indicated by white arrows. Ed/Rab11-positive compartments are present both in the medial cytoplasm and at the apical plasma membrane. (C) Quantification of the total intracellular Rab11 fluorescence per cell in control (RFP^+^) or *RASSF8* mutant (RFP^−^) cells. The *RASSF8* mutant values were normalised to the control values. Data are mean±s.e.m. *n*>44 cells from three different wings. Two-tailed Student's *t*-test: ****P<0.0001. (D) Quantification of the percentage of Ed colocalised with Rab11 per cell in control (RFP^+^) or *RASSF8* mutant (RFP^−^) cells. Data are mean±s.e.m. *n*>34 cells from three different wings. Two-tailed Student's *t*-test: ****P<0.0001. (E) Control wing. (E′) *nub-Gal4* driven *UAS-ed-RNAi* wing. (F) Quantification of relative wing roundness (ratio of AP to PD axis, normalised so that wild-type ratio=1). Data are mean±s.d. Two-tailed Student's *t*-test: ****P<0.0001. (G-J″) Hexagonal cell packing of control and *nub-Gal4*-driven *ed-RNAi* wings at 22, 26 and 30 h after puparium formation (APF). Confocal images of a region straddling the L3 vein (green rectangle in Fig. 1A) of control (G-G″) and *ed-RNAi* (I-I″) pupal wings stained using anti-Arm antibodies. Colour-coded images indicate the number of neighbours for each cell in control (H-H″) and *ed-RNAi* (J-J″). (K,L) Percentage of cells with four, five, six, seven or eight neighbours (colour coded as indicated) in control (K) and *ed-RNAi* (L) wings. The red line (octagons) is dashed so the green line (tetragons) can be seen. Data are mean±s.d. *n*=1600-4600 cells from four to eight individual wings. Scale bars: 10 μm in A-A‴,G-G″,I-I″; 2 μm in B-B″. See Table S2 for raw data.

Indeed, our RASSF8 two-hybrid screen identified the Baz N-terminus (amino acids 132-263) as a RASSF8 interaction partner. In S2 cell co-IP experiments, RASSF8 could associate with Baz (Fig. 4E), whereas ASPP could co-precipitate Baz only in the presence of RASSF8 (Fig. 4F). This indicates that the Magi/ASPP/RASSF8 complex can associate with Baz via a direct interaction between RASSF8 and Baz. Given the implication of Par3 as an exocyst receptor in mammalian cells (Ahmed and Macara, 2017), we tested whether loss of Baz leads to a mislocalisation of Rab11 vesicles in fly tissues. However, we observed only modest (1.1-fold) cytoplasmic Rab11 accumulation in the pupal wing (Fig. S4F,G) in *baz* mutant clones compared with 1.62-fold for *RASSF8* mutants (Figs 3E-E″ and 5A-C). This indicates that RASSF8 is required for trafficking of Rab11 vesicles independently of Baz in the pupal wing.

### Echinoid is a cargo of RASSF8/Sec15/Rab11 mediated transport

As our result suggested that RASSF8 is required for docking of Rab11 vesicles to the plasma membrane, we wished to identify the cargo(es) present in the stranded vesicles that accumulate in *RASSF8* mutant tissue. The exocyst has been implicated in Ecad trafficking in the fly notum (Langevin et al., 2005) and pupal wing (Classen et al., 2005), as well as in mammalian epithelial cells (Ahmed and Macara, 2017; Xiong et al., 2012; Yeaman et al., 2004). However, Ecad did not accumulate in intracellular vesicles in *RASSF8* mutant clones (Fig. S1F-F″). We examined the localisation of several transmembrane proteins involved in AJ maintenance and signalling (see Materials and Methods for details). The majority of these, such as the core PCP component Fmi (Fig. S4H-H″), were not affected. Using this candidate approach, we found that Echinoid (Ed) is accumulated in a punctate pattern in *RASSF8* clones at the AJs and in the cytoplasm (Fig. 5A-B″).

Ed is a large immunoglobulin (Ig) repeat trans-membrane homophilic adhesion molecule that cooperates with Ecad to mediate cell adhesion and sorting via the actomyosin network (Ho et al., 2010; Islam et al., 2003; Laplante and Nilson, 2006; Wei et al., 2005). Ed has functional similarities to mammalian nectins: both are junctional components that belong to the Ig superfamily and recruit the F-actin binding protein Canoe (afadin in mammals); however, but as their domain structure differs, Ed is considered to be nectin like rather than a nectin ortholog (Mandai et al., 2013; Wei et al., 2005). Ed has previously been observed to colocalise with early Rab5, late Rab7 and recycling Rab11 endosomal vesicles (Fetting et al., 2009; Li et al., 2015; Rawlins et al., 2003a). As Rab11 but not Rab5- or Rab7-positive vesicles accumulate in *RASSF8* mutant clones (Figs 3 and 4), we performed colocalisation analysis of Rab11 and Ed (Fig. 5B-D; see Materials and Methods). Confirming our previous results, we observed that Rab11 compartments accumulate in *RASSF8* mutant clones (Fig. 5C; 1.62-fold increase in cytoplasmic Rab11 compared with control). Furthermore, there was a significant increase in Rab11 and Ed colocalisation in *RASSF8* clones (Fig. 5D), suggesting that Ed trafficking by Rab11 is perturbed in *RASSF8* mutants. Ed was present in enlarged Rab11 compartments both in the cytoplasm and close to the apical plasma membrane, consistent with a failure to fuse with the junctions (Fig. 5B-B″).

We wished to test whether, like RASSF8, Ed is required for wing elongation and hexagonalisation. *ed* mutant clones trigger the formation of an acto-myosin cable in neighbouring wild-type cells, which often leads to their exclusion from the wing disc epithelium (Wei et al., 2005), making recovery of clones at the pupal stage difficult. However, we can partially inhibit Ed function in the wing blade by driving an RNAi construct under the *nubbin-GAL4* (*nub-GAL4*) driver. Similar to *RASSF8* mutants, *ed* depletion in the wing blade leads to an increase in the AP/PD ratio (Fig. 5E,F). Furthermore, we observed a defect in hexagonal packing in *ed*-depleted wings compared with control (at 30 h APF: control, 73%; *ed* depleted, 60%) (Fig. 5G-L). Thus, loss of Ed elicits similar pupal wing phenotypes to RASSF8 loss, consistent with the model that RASSF8 is required for Ed AJ trafficking during wing morphogenesis.

## DISCUSSION

The accurate and timely remodelling of epithelial tissues is a key feature of organogenesis (Harris and Tepass, 2010). Here, we explore the function of the RA domain-containing scaffold protein RASSF8 in epithelial morphogenesis using pupal wing development. We show that RASSF8 functions in this process independently of its partner ASPP, with which it regulates Src activity at the AJs (Langton et al., 2009) (Fig. S1). Our work reveals that RASSF8 is required for remodelling of the wing epithelium to a mature hexagonal lattice (Fig. 1, Fig. S1), a process dependent on planar polarised acto-myosin contractility and recycling of junctional components (Aigouy et al., 2010; Bardet et al., 2013; Classen et al., 2005; Gault et al., 2012; Ma et al., 2008; Warrington et al., 2013).

We identified the exocyst component Sec15 as a binding partner for RASSF8 (Fig. 2). As Sec15 is required for recycling of Ecad from the basal membrane back to the AJs in the pupal notum (Langevin et al., 2005) and inhibition of its binding partner Rab11 prevents pupal wing hexagonalisation (Classen et al., 2005), we investigated the consequences of RASSF8 loss on exocyst function. We found that Sec15 and Rab11, but not Sec5, accumulate in the cytoplasm of *RASSF8* mutant cells (Fig. 3), consistent with the idea that RASSF8 acts as an AJ receptor that allows exocyst-dependent docking of Rab11 vesicles prior to fusion with the target membrane.

### RASSF8-mediated trafficking of Echinoid vesicles

Although Ecad-positive REs accumulate in *sec15* mutant tissue in the pupal notum (Langevin et al., 2005), we observed no such accumulation in *RASSF8* mutant cells (Fig. 1, Fig. S1). This suggests that RASSF8 is not involved in trafficking of Ecad endosomes. Instead, we identified the Ig superfamily adhesion molecule Ed as a cargo whose delivery is dependent on RASSF8 (Fig. 5). The exocyst and Rab11 are involved in both biosynthetic and recycling trafficking (Heider and Munson, 2012), therefore RASSF8 could promote the delivery of newly synthesised and/or recycled Ed to the junctions. Ed depletion in the wing results in similar, although less pronounced, hexagonalisation and wing elongation defects to *RASSF8* mutants, suggesting that the *RASSF8* phenotype is at least in part due to defective Ed trafficking (Fig. 5). Interestingly, mammalian nectin-2α has been implicated in exocyst apical recruitment in MDCK cells (Yeaman et al., 2004), but ours is the first report of a nectin-like molecule as an exocyst cargo. With respect to the lack of Ecad cytoplasmic accumulation in *RASSF8* mutants, it is also worth noting that exocyst dependency of trans-membrane cargoes is tissue specific. For example, trafficking of the polarity protein Crumbs requires the exocyst in the embryonic epidermis (Blankenship et al., 2007; Roeth et al., 2009) and follicular epithelium (Aguilar-Aragon et al., 2020), but not in the pupal notum (Langevin et al., 2005), photoreceptors (Beronja et al., 2005) and renal tubules (Campbell et al., 2009).

How could disruptions in Ed trafficking lead to epithelial reordering defects? Like many Ig superfamily molecules, Ed can *trans*-dimerise (Islam et al., 2003; Rawlins et al., 2003a). Ed is also associated with the acto-myosin cytoskeleton via a direct interaction with the actin filament-binding protein Canoe (Wei et al., 2005). So far, the majority of Ed functions have been related to cell sorting at Ed expression boundaries. Indeed, at the boundary of *ed* mutant clones, Ed is lost from the junctions of wild-type cells that abut the mutant clones, inducing the assembly of a contractile acto-myosin cable that leads to apical constriction of the mutant cells (Wei et al., 2005). Acto-myosin contractility at the clone border, together with differential adhesion, leads to a cell-sorting phenotype characterised by a smooth border between the mutant and wild-type populations (Chang et al., 2011). Naturally occurring Ed expression boundaries can also trigger acto-myosin cable formation and drive cell-sorting events in several morphogenetic processes, such as dorsal closure (Laplante and Nilson, 2006; Lin et al., 2007), ommatidial rotation (Fetting et al., 2009; Ho et al., 2010) and ovarian follicle cell segregation (Laplante and Nilson, 2006). However, as we did not observe any Ed expression boundaries in the pupal wing, and *RASSF8* mutant clones do not display the characteristic round smooth border of *ed* mutant clones, the role of Ed in hexagonalisation is likely to be distinct. Whether this role involves cytoskeletal modulation or an Ed adhesive function through homophilic association or heterophilic interactions with other partners, such as its paralog Friend of Echinoid (Özkan et al., 2013) remains to be investigated. Alternatively, as Ed has been shown to modulate several signalling pathways, such as Notch (Chandra et al., 2003; Escudero et al., 2003; Rawlins et al., 2003a), Hippo (Yue et al., 2012) and Epidermal growth factor receptor (Bai et al., 2001; Fetting et al., 2009; Islam et al., 2003; Rawlins et al., 2003b; Spencer and Cagan, 2003), it may be acting via cell-cell signalling.

### Junctional targeting of the exocyst

Structural analyses of the yeast exocyst have shown that the full octameric complex can be subdivided into two distinct subcomplexes, with subcomplex I composed of Sec3, Sec5, Sec6 and Sec8, while complex II contains Sec10 and Sec15, and Exo70 and Exo84 (Ganesan et al., 2020; Heider et al., 2016; Mei et al., 2018). Macara and colleagues have recently shown that, in mammalian cells, the two subcomplexes can arrive at the plasma membrane following different kinetics, suggesting that these can be recruited to the target membrane via independent mechanisms (Ahmed et al., 2018). Our data indicate that RASSF8 loss disrupts the localisation of Sec15 (subcomplex II), while Sec5 (subcomplex I) is not affected (Figs 3 and 4). Interestingly, numerous lines of evidence show that subcomplex II plays a key role in exocyst targeting to the adherens/tight junctions. Indeed, binary associations between Exo70 and Par3 (Ahmed and Macara, 2017), between Sec10 and Par6 (Zuo et al., 2011) or Armadillo/β-catenin (Langevin et al., 2005), between Exo84 and Par6 (Das et al., 2014), and between Sec15 and RASSF8 (this study) have been reported. This diversity of exocyst recruitment mechanisms may reflect the diverse nature of cell-cell contacts across different tissues and developmental stages. In the pupal wing, RASSF8 appears to play a more essential role in Rab11 vesicle trafficking than its binding partner Baz/Par3 (Fig. 5, Fig. S4), but it would be interesting to determine whether the different exocyst subcomplex II/junctional component interactions are differentially required according to cell type, context and cargo. Understanding how specific interactions of the exocyst with target membranes ensures accurate sorting of adhesion molecules to the appropriate subcellular localisation in the correct spatial and temporal pattern is key to understanding how epithelial tissues are built, remodelled and maintained.

## MATERIALS AND METHODS

### *Drosophila* stocks

*FRT82B Sec15^1^* was a gift from Hugo Bellen (Baylor College of Medicine, Houston, TX, USA) (Mehta et al., 2005), *DEcad::GFP* was a gift from Yang Hong (University of Pittsburgh Medical School, PA, USA) (Huang et al., 2009) and *baz^eh747^ FRT19A* was a gift from Andreas Wodarz (University of Cologne Medical School, Germany) (Eberl and Hilliker, 1988). *RASSF8^6^* (Langton et al., 2009) and *ASPP^8^* (Langton et al., 2007) have been previously described. *ubi-Sec15::GFP* transgenic flies were generated by introducing the Sec15 gene into a modified pKC26 plasmid containing the *ubiquitin-63E* promoter and a C-terminal GFP tag (Zaessinger et al., 2015). This vector was injected by Bestgene into flies bearing a 3L *attP* landing site (VIE-217). *ed-RNAi* (BL-38243) and *Df(3R)BSC321* (BL-24909) were obtained from the Bloomington *Drosophila* Stock Center.

### Genotypes

The following genotypes are shown in the figures.

Fig. 1B, Fig. S4A-A″: *w^iso^*

Fig. 1B′: *w;; RASSF8^6^*

Fig. 1B″: *w;; RASSF8^6^*/*Df (3R)BSC321*

Fig. 1C-D″, Fig. S1A-B″,G-H″: *w; Ecad::GFPki*

Fig. 1F-G″, Fig. S1C-D″: *w; Ecad::GFP; RASSF8^6^/RASSF8^6^*

Fig. 3A-C‴: *hsFlp;; FRT82B ubi nlsRFP/ubi-Sec15::GFP, FRT82B RASSF8^6^*

Fig. 3D-F″ *hsFlp;; FRT82B ubiGFP/FRT82B RASSF8^6^*

Fig. 4A-A″: *hsFlp;; FRT82B ubiRFP/FRT82B Sec15^1^*

Fig 4B-B″, Fig 5A-B″: *hsFlp;; FRT82B ubi nlsRFP/FRT82B RASSF8^6^*

Fig. 4C-D′, Fig. S3A-C″: *eyFlp;; FRT82B ubiGFP/ FRT82B RASSF8^6^*

Fig. 5E,G-H″: *w; nub-Gal4/UAS-RFP;*

Fig. 5E′, 5I-J″: *w; nub-Gal4/UAS-ed RNAi (TRiP.HMS01687);*

Fig. S1I-J″: w; *Ecad::GFP, ASPP^8^;*

Fig. S1F-F″, Fig. S4H-H″: *hsFlp; Ecad::GFPki/+; FRT82B ubi myr-RFP/FRT82B RASSF8^6^*

Fig. S4F-F″: *baz^eh747^ FRT19A/ubi-RFP, hsFLP FRT19A*

### Yeast two-hybrid

The yeast two-hybrid screen with full-length RASSF8 cloned as an N-terminal LexA fusion in pB29 as bait was performed by Hybrigenics (Paris, France) using a *Drosophila* whole-embryo cDNA collection (RP2).

### Plasmid construction

The *Sec15* gene was amplified from DGRC cDNA clone RE55430. Genes of interest were cloned into Gateway entry vector and subsequently expression vectors containing HA/Myc tags (*Drosophila* Gateway Vector Collection - http://emb.carnegiescience.edu/labs/murphy/Gateway%20vectors.html).

Small GTPases used for *in vitro* GST pulldown assay were reverse transcribed from total mRNA isolated from wild-type adult flies, cloned into the pGEX4T-1 vector and verified by sequencing.

### GST fusion protein expression

Small GTPases were expressed in BL21(DE3)pLysS bacteria (Promega). Protein expression was induced with 0.5 mM isopropyl β-D-1-thiogalactopyranoside (IPTG) and carried out at 18°C overnight. Bacteria were lysed by sonication in LyBTL [50 mM Tris HCl (pH 7.5), 50 mM NaCl, 5 mM MgCl_2,_ 0.1% (v/v) Triton X-100] buffer containing 1 mM DTT, 1 mM PMSF, 0.5 g/l lysozyme and protease inhibitor cocktail (Roche). The supernatant was incubated with glutathione Sepharose 4B beads (GE Healthcare) at 4°C for 1 h.

### *In vitro* binding assay for small GTPases

Glutathione sepharose beads with 60 μg small GTPases were loaded with GTPγS or GDP in GTPase loading buffer [20 mM HEPES (pH 7.5), 25 mM NaCl, 10 mM EDTA, 2 mM GTP/ GTPγS] for 20 min at 37°C. NL100 buffer [20 mM HEPES (pH 7.5), 100 mM NaCl, 5 mM MgCl2, 0.1% (v/v) Triton X-100] containing 0.1 mM GTPγS or GDP was added immediately afterwards to stop nucleotide exchange. S2 cell lysate was added to the beads in NL100 buffer. The binding was performed at 4°C for 1 h.

### Western blotting and co-IP assays

S2-DGRC cells (Cellosaurus CVCL_TZ72) were obtained from the *Drosophila* Genomics Resource Center and transfected with Effectene (Qiagen). For co-IP assays, cells were lysed in HEPES lysis buffer [50 mM HEPES (pH 7.4), 150 mM NaCl, 0.5% (v/v) Triton X-100] supplemented with phosphatase inhibitor cocktails 1 and 2 (Sigma) and Protease Inhibitor Cocktail (Roche) on ice for 15 min. Soluble cell lysates were obtained after centrifugation at 15,000 ***g*** for 15 min at 4°C. Protein concentrations were determined using the Dc protein assay (Bio-Rad). Lysates were then incubated with Protein A/G sepharose beads and appropriate antibodies for 2 h at 4°C. Immunoprecipitates were then purified after washing four times with HEPES lysis buffer. Detection of purified proteins and associated complexes was performed by immunoblot analysis using chemiluminescence (GE Healthcare). Western blots were probed with anti-FLAG (mouse M2, Sigma; 1:1000), anti-Myc (mouse 9E10, Santa Cruz Biotechnology; 1:1000), anti-HA (rat 3F10, Roche; 1:1000) and anti-RASSF8 (Langton et al., 2009) antibodies.

### Electron microscopy and image analysis

Dissected pupal retinas were fixed in 4% formaldehyde and 1.5% glutaraldehyde in 0.1 M phosphate buffer for 1 h at room temperature. Samples were further fixed with reduced osmium tetroxide for 1 h followed by 1% tannic acid in 0.05 M sodium cacodylate for 45 min. Samples were then dehydrated through a graded series of ethanol, embedded in Epon resin and sectioned at 70 nm using an Ultracut UCT ultramicrotome (Leica Microsystems) and post-stained with lead citrate. Images were obtained with a Tecnai G^2^ Spirit transmission electron microscope (FEI Company) and an Orius CCD camera (Gatan). Images of ommatidia were taken from the apical plane. Non-overlapping images from a single plane were used to quantify gaps in the cell-cell junctions (*n*=17) using manual segmentation in Amira software (Visage Imaging). A projection of the segmented gaps from overlaid, but non-sequential, images illustrates the increase in gaps in the mutant over the wild type.

### Quantification of the number of neighbours of each cell in pupal wing

Z projections of pupal wings labelled with Ecad::GFP or fluorescently labelled anti-Arm antibodies were created by ImageJ. The number of neighbours of each cell was quantified using the Packing Analyzer (v2.0) described previously (Aigouy et al., 2010).

### Quantification of Ed and Rab11 colocalisation

Quantification of Rab11 expression or colocalisation of Ed with Rab11 was achieved using a FIJI Plugin called Particle Mapper: https://github.com/djpbarry/CALM/wiki/Particle-Mapper (see Wanaguru et al., 2018 for details). However, because Particle Mapper requires a nuclear marker as one of its inputs, additional processing was required in order to generate a pseudo-nuclear channel for the purposes of this study.

Pseudo-nuclear markers were generated as follows. Multi-channel confocal image stacks were first analysed to identify and isolate the highest contrast *z*-position. Subsequently, the Echinoid channel at this *z*-location was isolated and noise and background were suppressed. Grey-level thresholding was then used to generate a binary image, which was subsequently skeletonised and pruned. The resultant inter-skeleton regions, following an erosion operation, were assumed to be reasonable approximations of the cell interiors. A FIJI script to automate all of these steps is available online: https://github.com/djpbarry/wing-cell-quant/blob/main/Rab11_quant.cppipe.

For the data presented in this paper, the script default options were used. At least 31 cells per genotype were analysed from three different retinas. The analysis was carried out in single confocal sections (0.5 μm in depth) at the level of the adherens junctions.

### Quantification of Ecad at cell junctions

Quantification of Ecad intensity at cell-cell junctions was achieved using a combination of a FIJI script (https://github.com/djpbarry/wing-cell-quant/blob/main/Blob_Detector.ijm) and a CellProfiler pipeline (https://github.com/djpbarry/wing-cell-quant/blob/main/Ecad%20in%20F8%20clones.cppipe).

Briefly, the locations of cell centres were estimated in FIJI using a blob detection approach based on calculation of Hessian eigenvalues: https://imagescience.org/meijering/software/featurej/hessian.

These centre locations were then used to seed a full cell segmentation using the marker-controlled watershed in MorphoLibJ (Legland et al., 2016).

The resultant cell segmentations were then analysed using the CellProfiler pipeline. Cells were first filtered based on size and Ecad intensity to remove those likely to be coincident with veins, which have elevated Ecad density and could have biased the results. The Ecad intensity at the cell periphery was then quantified per cell across wild-type and *RASSF8* mutant populations.

### Analysis of *Drosophila* wing roundness

For analysis of wing roundness, young adult wings were processed, mounted and imaged as described previously (Ribeiro et al., 2010). The roundness of the wing is defined by the length of AP axis along the L3 vein divided by the length of the PD axis crossing the posterior crossvein.

### Genetics and immunochemistry

Mosaic tissues were obtained using the FLP/FRT system with *hsFlp*. Flies were heat-shocked for 60 min at 48 h and 72 h after egg deposition. Pupae were staged by collecting white prepupae 3 days after the first heat-shock and incubating at 25°C for the indicated times. Pupal wings and retinas were fixed in 8% and 4% paraformaldehyde in PBS, respectively, for 30 min (larval wing discs in 4% paraformaldehyde in PBS for 30 min), washed three times with PBS, permeabilised with PBT (PBS+0.3% Triton x100), blocked with PBT+1% BSA, and immunostained using the indicated primary antibodies in PBT+1% BSA at 4°C overnight and secondary antibodies for 2 h at room temperature.

Primary antibodies used were rabbit anti-Rab5, anti-Rab7, anti-Rab11 (1:2000, gifts from Akira Nakamura, Kumamoto University, Japan; Tanaka and Nakamura, 2008), guinea pig anti-Sec15 and anti-Hrs (1:1000, a gift from Hugo Bellen; Lloyd et al., 2002; Mehta et al., 2005), rat anti-Ed (1:1000, a gift from Jui-Chou Hsu, National Chiao Tung University, Taiwan; Wei et al., 2005), mouse anti-Sec5 (22A2) antibody (1:200, a gift from Thomas Schwartz, Harvard Medical School, Boston, MA, USA; Murthy et al., 2003). The rat anti DE-cadherin (1:100, developed by T. Uemura, Kyoto University, Japan), mouse anti-Arm (1:100, developed by E. Wieschaus, Princeton University, NJ, USA) and anti-Fmi #74 (1:20, developed by T. Uemura) were obtained from the Developmental Studies Hybridoma Bank. Secondary antibodies used were rhodamine red-X donkey anti-rabbit, anti-rat and anti-mouse, fluorescein isothiocyanate (FITC) donkey anti-rabbit, anti-rat and anti-mouse (Jackson ImmunoResearch), goat anti-rat Alexa 647, and goat anti-rabbit Alexa 633, all at 1:500. Fluorescence images were acquired with a Zeiss LSM780 or a Zeiss LSM880 confocal.

Antibodies against transmembrane proteins tested for accumulation in *RASSF8* mutant clones were: mouse anti-Fmi (1:20, DSHB Flamingo #74), rabbit anti-Ds (1:1000, from David Strutt, University of Sheffield, UK; Strutt and Strutt, 2002), mouse anti-Roughest/IrreC (1:10, from Karl-Friedrich Fischbach, Albert-Ludwigs-University Freiburg, Germany; Schneider et al., 1995), mouse anti-Notch-ICD (1:100, DSHB C17.9C6; deposited by S. Artavanis-Tsakonas, Harvard Medical School, Boston, MA, USA), rat anti-Crb (1:1000, from Franck Pichaud, University College London, UK; Walther et al., 2016), rat anti-Fat (1:1000, from Helen McNeill, Washington University School of Medicine, St Louis, MO, USA), rabbit anti-EGFR (1:1000, from Erika Bach, New York University Grossman School of Medicine, USA), rat anti-SNS (1:1000, from Susan Abmayr, Stowers Institute for Medical Research, Kansas City, MO, USA; Bour et al., 2000), rat anti-Hibris (1:1000, from Tetsuya Tabata, The University of Tokyo Bunkyo-ku, Japan; Sugie et al., 2010) and rat anti-Kirre (1:1000, from Susan Abmayr; Galletta et al., 2004).

### Statistics

Statistical analysis was performed using Prism (GraphPad Software). All raw data and details of statistical tests are in Tables S1 and S2. All averages correspond to mean.

## Supplementary Material

Supplementary information

Reviewer comments
