# Supplementary material for: RASSF8-mediated transport of Echinoid via the exocyst promotes Drosophila wing elongation and epithelial ordering
Source: Development. 2021 Oct 14;148(20):dev199731. doi: 10.1242/dev.199731 (PMC8572004; doi:10.1242/dev.199731)
Supplement: Supplementary information [file develop-148-199731-s1.pdf]

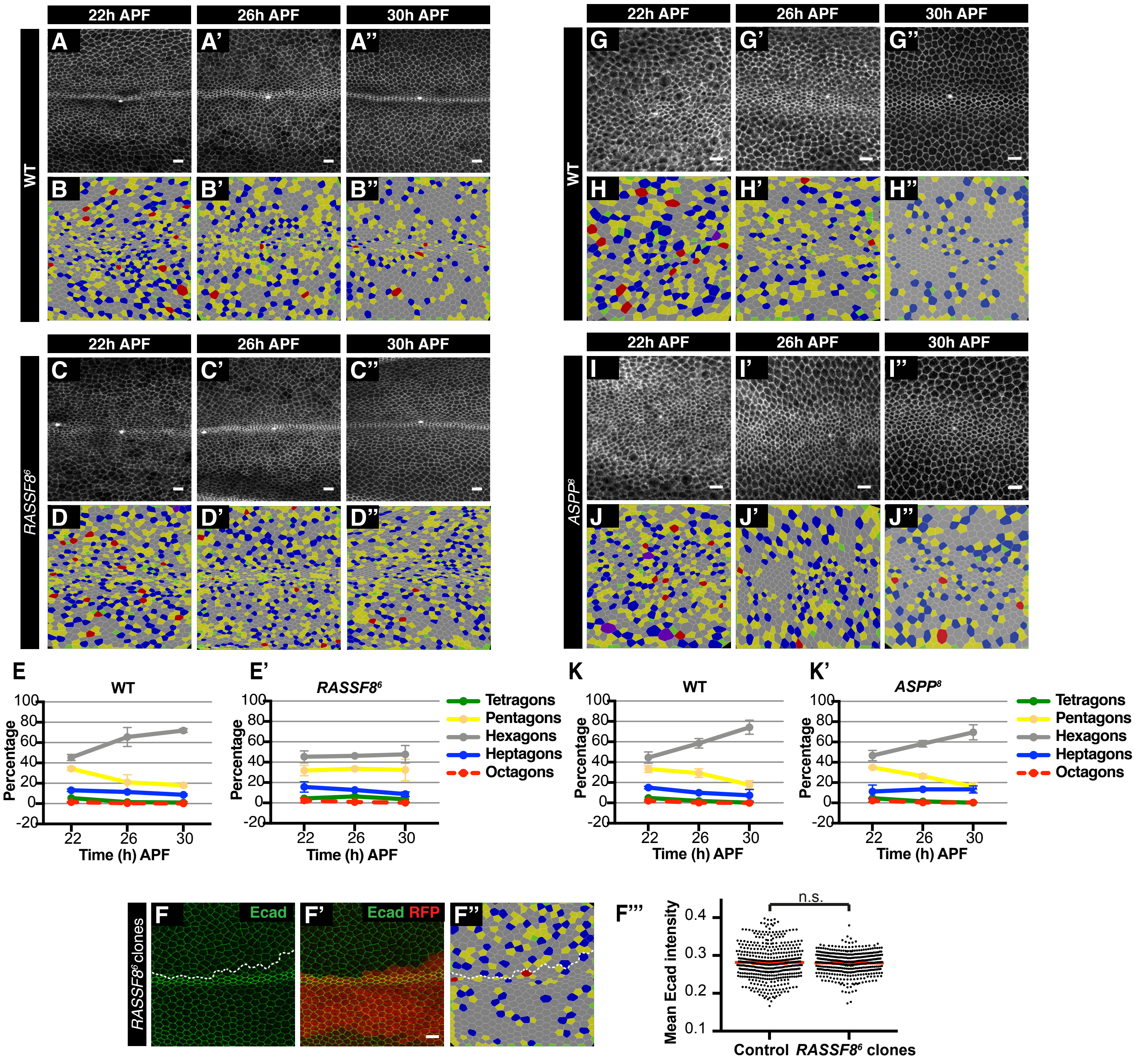

Development • Supplementary information

Fig. S1. Wing cell packing defects in *RASSF8* mutants

(A-D'') Confocal images of Ecad::GFP-labelled wild type (A) and *RASSF8* mutant (C) pupal wings in a region straddling the L3 vein (green rectangle in Fig. 1A) at 22, 26, 30 hours APF. Colour-coded images indicate the number of neighbours for each cell in wild type (B) and *RASSF8* mutant (D). (E-E') Percentage of cells with four, five, six, seven or eight neighbours (colour coded as indicated) in wild type (E) and *RASSF8* mutants (E') (n=2500-5000 cells from 3 to 5 individual wings; error bars = s.d.). (F-F'') *RASSF8* mutants alter hexagonal cell packing cell autonomously. Ecad::GFP and merged images of *RASSF8* mutant clones marked by the absence of RFP at 36 hours APF. Clone boundaries are marked by white dotted line. (F''') Quantification of average Ecad::GFP intensity per cell at the cell junctions in control and *RASSF8* mutant cells. Error bars = s.e.m.; n=415-451 cells from 3 different wings. Two-tailed Student's t-test: n.s.=not significant (p=0.67). (G-J'') Confocal images of Ecad::GFP-labelled wild type (G) and *ASPP* mutant (I) pupal wings in a region straddling the L3 vein (green rectangle in Fig. 1A) at 22, 26, 30 hours APF. Colour-coded images indicate the number of neighbours for each cell in wild type (H) and *ASPP* mutant (J). (K, K') Percentage of cells with four, five, six, seven or eight neighbours (colour coded as indicated) in wild type (K) and *ASPP* mutants (K'). The red line (octagons) has been dashed so the green line (tetragons) can be seen. n=1400-3000 cells from 3 to 8 individual wings; error bars = s.d. Scale bars: 10 µm. See Table S1 for raw data.

Development • Supplementary information

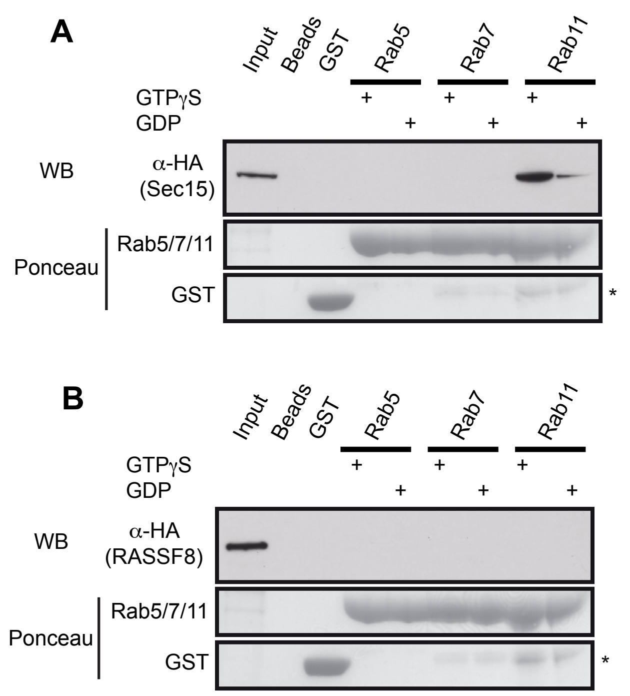

Fig. S2. RASSF8 does not directly bind to Rab11

(A, B) GST-pulldown experiments using GST-Rab5 (early endosomes), Rab7 (lysosomes) and Rab11 (recycling or biosynthetic endosomes) with (A) Sec15 or (B) RASSF8. Rab family GTPases were loaded with GTPγS or GDP. As controls, Glutathione beads and GST-only pulldowns were used. Equal protein levels of small GTPases and GST were verified by Ponceau S staining (middle and bottom panels). Asterisks mark degradation products of the small GTPases.

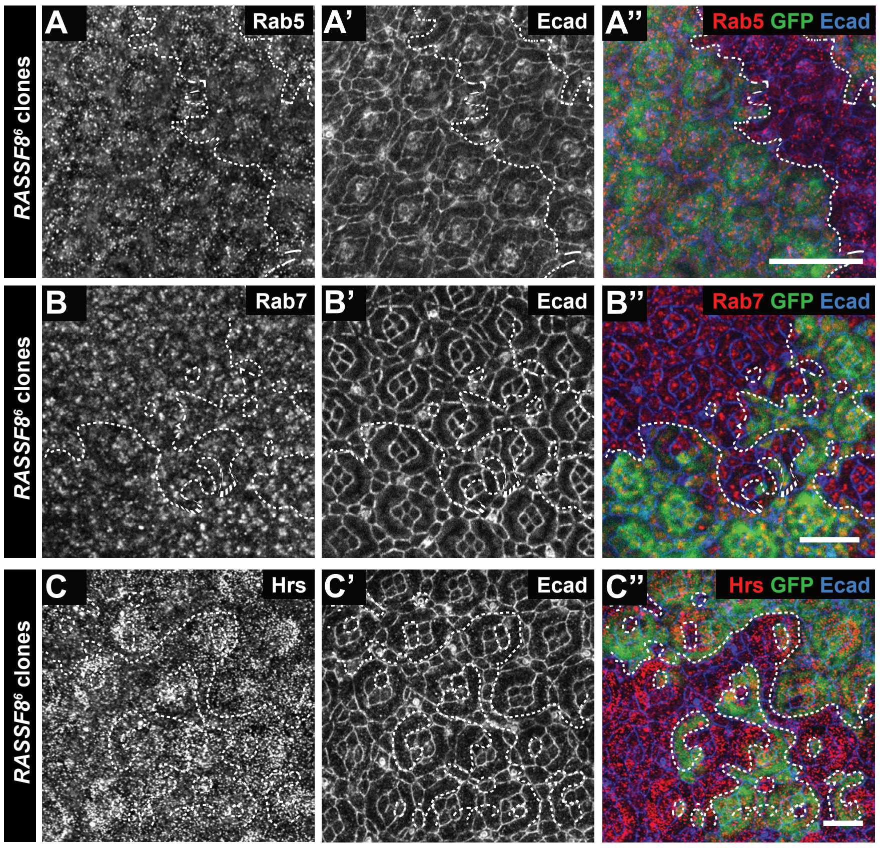

Fig. S3. Normal Rab5, Rab7 and Hrs localisation in *RASSF8* retinal clones

(A-C'') Confocal micrographs of pupal retinas at 26 hours APF bearing *RASSF8* mutant clones generated using *eyFLP* and stained as indicated. White dotted lines label the clone boundaries. Staining for Rab5 (A-A''), Rab7 (B-B'') and Hrs (C-C'') is not altered in *RASSF8* mutant pupal retina clones marked by the absence of GFP. Scale bars: 10 µm.

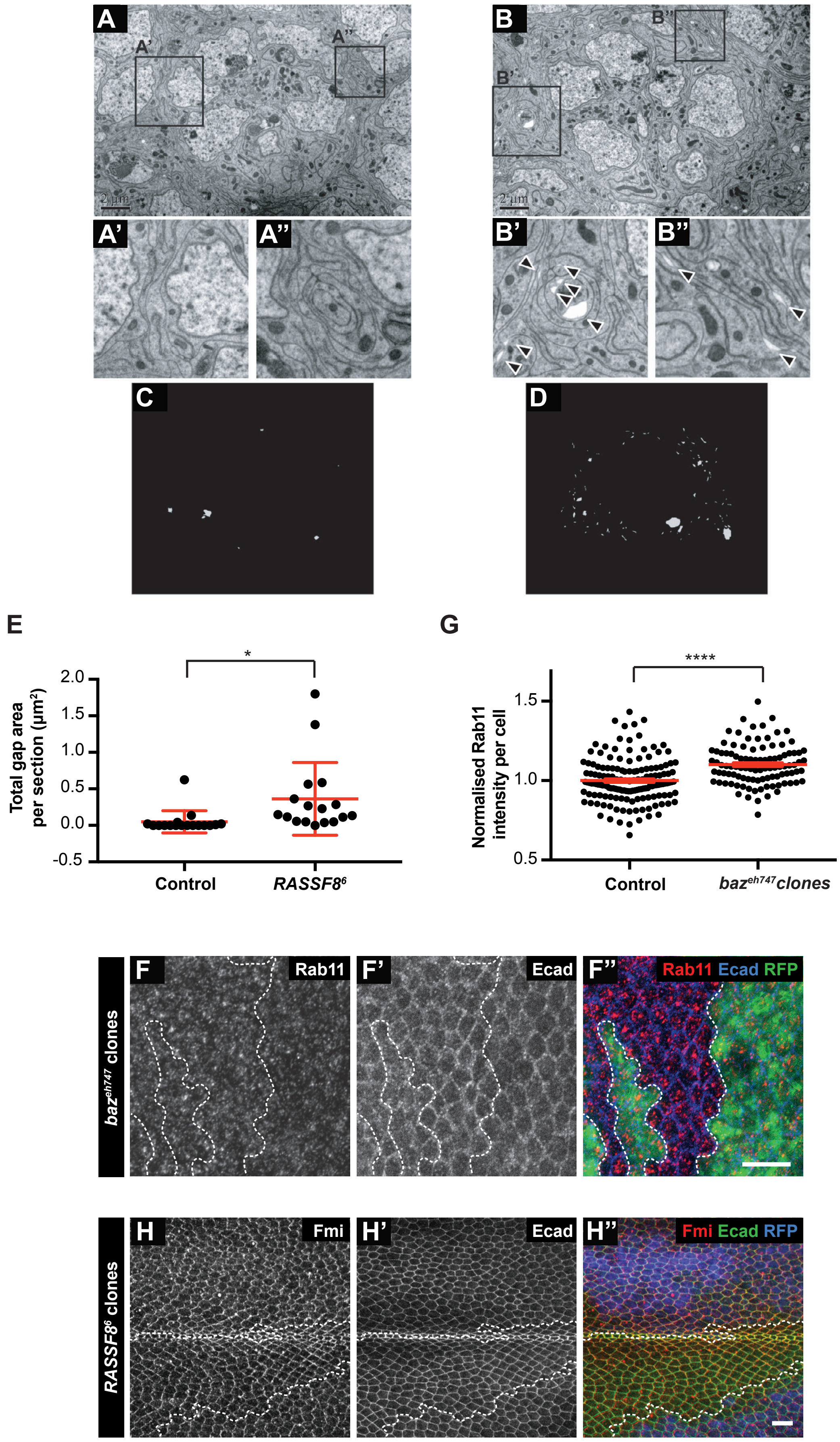
A
A'
A''
2 µm
B
B'
B''
2 µm
A'
A''
B'
B''
C
D
E
*
2.0
1.5
1.0
0.5
0.0
-0.5
Total gap area
per section (µm²)
Control
RASSF8⁶
G
****
1.5
1.0
0.5
Normalised Rab11
intensity per cell
Control
baz^eh747 clones
F
Rab11
F'
Ecad
F''
Rab11 Ecad RFP
baz^eh747 clones
H
Fmi
H'
Ecad
H''
Fmi Ecad RFP
RASSF8⁶ clones

Fig. S4. Intercellular gaps in *RASSF8* mutant pupal retinas

Electron micrographs reveal a defect in cell-cell adhesion in *RASSF8* mutant retinas at 26 hours APF. TEM of control (A) and *RASSF8* mutant (B) ommatidia at the level of the apical AJs reveals an increase in gaps at cell-cell junctions. Inserts of representative areas show an absence of gaps in control (A'-A'') and numerous gaps in the mutant (arrows) (B' and B''). The gaps in ommatidia from single plane images (n = 17 images) were quantified by manual segmentation in Amira. The resulting segmentations are shown as a projection of the non-sequential overlaid images for control (C) and mutant (D). (E) Quantification of gap area per section in control and *RASSF8* mutant retinas. Error bars = s.d.; n=17. Two-tailed Student's t-test, * p = 0.0186. Scale bars in A and B: 2 µm. (F-F") Staining for Rab11 (red) is only modestly increased in *baz* mutant pupal wing clones marked by the absence of RFP (green). Ecad staining is in blue. (G) Quantification of the total intracellular Rab11 fluorescence per cell in control (RFP+) or *baz* mutant (RFP-) cells. The *baz* mutant values were normalised to the control values. Error bars = s.e.m.; n=101-149 cells from 3 different wings. Two-tailed Student's t-test: **** p<0.0001. Scale bar: 10 µm. See Table S2 for raw data. (H-H'') *RASSF8* mutant pupal wing clones at 30 hours APF marked by absence of GFP (green stained with anti-Fmi antibody. Polarised staining of Fmi is normal in RASSF8 and is concentrated on the proximal and distal cell boundaries.

Table S1. Raw data and statistics for Figures 1 and S1

Click here to download Table S1

Table S2. Raw data and statistics for Figures 5 and S4

Click here to download Table S2

Development • Supplementary information
