# Supplementary material for: RASSF8-mediated transport of Echinoid via the exocyst promotes Drosophila wing elongation and epithelial ordering
Source: Development. 2021 Oct 14;148(20):dev199731. doi: 10.1242/dev.199731 (PMC8572004; doi:10.1242/dev.199731)
Supplement: Reviewer comments [file develop-148-199731_review_history.pdf]

RASSF8-mediated transport of Echinoid via the exocyst promotes Drosophila wing elongation and epithelial ordering

Eunice Ho Yee Chan, Yanxiang Zhou, Birgit L Aerne, Maxine V Holder, Anne Weston, David J Barry, Lucy Collinson and Nicolas Tapon

DOI: 10.1242/dev.199731

Editor: Thomas Lecuit

Review timeline

Original submission:	22 April 2021
Editorial decision:	11 June 2021
First revision received:	25 August 2021
Accepted:	13 September 2021

Original submission

First decision letter

MS ID#: DEVELOP/2021/199731

MS TITLE: RASSF8-mediated recycling of the nectin Echinoid via the exocyst promotes Drosophila wing elongation and epithelial ordering

AUTHORS: Eunice Ho Yee Chan, Yanxiang Zhou, Birgit L Aerne, Maxine V Holder, Anne Weston, David J Barry, Lucy Collinson, and Nicolas Tapon

I have now received all the referees reports on the above manuscript, and have reached a decision. The referees' comments are appended below, or you can access them online: please go to BenchPress and click on the 'Manuscripts with Decisions' queue in the Author Area.

The overall evaluation is very positive and we would like to publish a revised manuscript in Development, provided that the referees' comments can be satisfactorily addressed. Please attend to all of the reviewers' comments in your revised manuscript and detail them in your point-by-point response. If you do not agree with any of their criticisms or suggestions explain clearly why this is so.

We are aware that you may currently be unable to access the lab to undertake experimental revisions. If it would be helpful, we encourage you to contact us to discuss your revision in greater detail. Please send us a point-by-point response indicating where you are able to address concerns raised (either experimentally or by changes to the text) and where you will not be able to do so within the normal timeframe of a revision. We will then provide further guidance. Please also note that we are happy to extend revision timeframes as necessary.

Reviewer 1

Advance summary and potential significance to field

Chan et al. report on the function of RASSF8 in permitting the proximo-distal elongation of the Drosophila wing. They perform a 2-hybrid screen to identify interacting proteins, and find that the exocyst subunit Sec15 can directly interact with RASSF8, which is also nicely confirmed in vivo in S2

© 2021. Published by The Company of Biologists under the terms of the Creative Commons Attribution License (https://creativecommons.org/licenses/by/4.0/).

cells. The authors then go on to test if membrane trafficking is altered in RASSF8 compromised cells, and find that recycling endosomes accumulate – this matches expectations from prior knowledge of what occurs when exocyst complex function is disrupted. They then go on to try to identify the membrane cargo that is delivered through a RASSF8-Sec15/exocyst complex-Rab11 pathway, and suggest that Echinoid may be the relevant cargo (a bit of a surprise given previous work implicating E-cad). However, moderate loss of function (shRNA) of Echinoid nicely phenocopies RASSF8 disruption. The authors also show that RASSF8 has a similar function in the fly eye, and test alternate hypotheses around Par-3, ASPP, and Magi. In general, this is an exceptionally well-done study – the authors are careful and the work is performed at a high level. The paper is nicely written (Introduction is beautiful and comprehensive), it logically flows, and the authors thoroughly think through alternate possibilities (each time I started to think “what about this” the authors preemptively addressed it). In my opinion, this should make a very nice addition to Development. I have only a few additional comments:

Comments for the author

1) page 8, “Interestingly, the localisation of Sec5, which is primarily cortical in the pupal wing, is not altered in RASSF8 mutant clones, suggesting that RASSF8 loss only affects vesicle-associated exocyst components or a subset of Sec15 exocyst complexes not associated with Sec5 (Figure S3B-B’).” It seemed more likely to me that this would suggest that Sec5 is not sensitive to Sec15 loss, also in keeping with the subcomplex data from Macara.

2) It would be nice to have some quantitation of Ed and E-cad levels at the plasma membrane (levels actually look higher in the clonal region?). Or, alternatively, if the authors think the central phenotype is Ed breaks could continuity be assayed (standard deviation of fluorescent intensities across an interface, for example)?
This was the one part of the study that led to questions for me – given prior work and also given that many TM proteins would be expected to transit through REs, a little more clarity on the subject would be good.
However, the Ed phenocopy of RASSF8 was nice. Not all data needs to fit perfectly, just was the one curious result in the paper for me.

3) A small item, has moderate Crumbs loss been looked at at these stages? It is another important TM protein that has been implicated in being trafficked through exocyst and Rab11 trafficking.
Compliments to the authors – a great job on a near-perfect first submission. Very impressive.

<u>Reviewer 2</u>

Advance summary and potential significance to field

Chan and colleagues continue the analysis of RASSF8 that the Tapon lab identified as a adherens junction associated protein a few years back. RASSF8 was shown to form a complex with ASPP and Magi and to contribute to the junctional localization or regulation of the polarity protein Par3 and Src regulating kinase Csk. The authors noticed that RASSF8 mutant flies have shorter and broader wings suggesting defects in cell rearrangements and cell ordering during pupal wing development, a conclusion confirmed by live imaging of pupal wings. To further explore the function of RASSF8 and to identified the underlying mechanism of RASSF8 function in wing development the authors carried out a yeast-2-hybrid screen and identified Sec15 as a binding partner for RASSF8. Biochemical approaches suggest that RASSF8 and Sec15 interact directly.
Sec15 is a component of the exocyst complex that acts in conjunction with Rab11 to transport cargo to the junction in epithelial cells. Rab11 and Sec15 show increased cytosolic accumulation in RASSF8 mutant cells suggesting defects in surface delivery of vesicles. In examining several potential cargo proteins the authors identify the nectin-like junctional protein Echinoid as a relevant cargo, which co-accumulates with Rab11 in cytosolic vesicles in RASF8 mutant cells, and when removed displays the same defects in cell ordering and and adult wing shape as seen in RASSF8 mutants. The authors conclude that RASSF8 mediate the association of Sec15 (and the exocyst) with specific Rab11-positive (recycling?) endosomes to facilitate surface transport of Echinoid. Interestingly, the same endosomes to apparently not transport E-cadherin or Crumbs, suggesting an Echinoid-specific RASSF8-dependent vesicle population.

© 2021. Published by The Company of Biologists under the terms of the Creative Commons Attribution License (https://creativecommons.org/licenses/by/4.0/).

This is a nice paper that enhances our understanding of the mechanisms underpinning the dynamics and function of adherens junction in an important model for cell rearrangement and epithelial ordering. This paper is very much suitable for Development; given that there are 4 Figures, the editor may decide to suggest a Report format for publication.

Comments for the author

I have a few minor points:
1) Papers 1-4 are all old reviews. Perhaps the authors could update their reading list.

2) Rab11 could mediate recycling or biosynthetic trafficking (as it does for example for E-cadherin in MDCK cells: Lock and Stow, MBC 2005). The authors should add evidence Ed recycling or biosynthetic transport or discuss these option appropriately.

3) Hrs is not a lysosome marker. It is part of ESCRT-0 and a marker of 'mature endosomes' and detaches from the late endosomes before they reach the lysosome state.

4) Ed may also been a 'homotypic' adhesion molecules (which means it mediates adhesion between the same cell types) but the author presumably mean 'homophilic' adhesion molecule (meaning Ed on one cells binds to Ed on the adjacent cell). Ed is not a clear nectin homolog. Perhaps best to identify it as a nectin-like protein in term as AJ localization and binding to Afadin/Canoe.

Reviewer 3

Advance summary and potential significance to field

Promoting tissue morphogenesis without compromising tissue integrity depends on dynamic reorganization of adherens junctions. Here the authors describe a novel function of the RA-domain protein RASSF8 as a player in AJ component exocytosis and overall tissue dynamics. Building on earlier work on the function of the RASSF8-ASPP complex in the tissue specific regulation of junctional components like Magi and Bazooka, they provide convincing evidence for an ASSP-independent role of RASSF8. They show that RASSF8 acts as an anchor for recycling endosomes that deliver actomyosin regulating factors to the junctions. They make use of the developing Drosophila wing to study tissue and junction dynamics in vivo by combining qualitative and quantitative approaches. Furthermore, they combine their interesting findings with biochemical protein-interaction approaches. The data is overall well presented and well explained and will be interesting for a broad readership interested in both tissue and junction dynamics and also intracellular trafficking. The study provides further insights into these processes with are crucial for development but about which questions remain. We have some relatively minor suggestions for how the presentation of the data could be clarified, and how amplifying on some points would strengthen the story.

Comments for the author

Major comments:
1. The central point of the paper concerns role of Rassf8 in localization/function of the exocyst and Rab11. They find striking effects. However, two issues detract from careful analysis of the data. Overall the use of grey scales for single channel images would improve the presentation of the images—this is true in Figures 3 and 4 and in several of the Suppl. Figures. Second, they have images of sufficient quality that one could examine subcellular localization, but the magnifications used preclude this. They need to include closeups of images like those in Figure 3A-F—they do provide one example in Figure 4B. This was out most important issue with the data. On a more minor point, tell us in the main text whether the Sec15:GFP used was an insertion at the endogenous locus or an overexpressed protein, and give us a clearer indication of how +/+ tissues were distinguished from +/- tissues in Figure 3.

2. Concerning Figures 1B-G, 4G-L, S1A-K: As indicated in the text, these images and quantifications were taken at different positions in the wing, an overview image of the wing with a

© 2021. Published by The Company of Biologists under the terms of the Creative Commons Attribution License (https://creativecommons.org/licenses/by/4.0/).

depiction of the actual position would be helpful. Also, a legend of the axes within wing images would be helpful.

3. The text describing the results of the cell packaging could make clearer that the packaging represents the number of neighbors of a single cell.

4. Results concerning Fig S3A/ Sec5 in RASSF8 clones: In the text it is stated, that the Sec5 amount in clones is not altered, however this is not really clear in the image. The conclusion would benefit from a quantification/line plot.
The same is true for Figure S4F/ the statement, that there is no change in Ecad and Rab11 levels in baz clones.

5. Figure S4A-E/ Holes in retina junctions: Is a similar disruption of junctions/ fragmentation of junctional markers detectable in wing tissues? And if not, is this due to a RASSF8-Sec15 independent function (as f. e. RASSF8-ASPP-Magi) that may not play a role in wing development?

6. It is stated several times, that the planar polarization/redistribution of junctional components is crucial for the probed tissue dynamics. Have they thought about the localization of planar polarized proteins in RASSF8 mutant tissues? And is Ed planar polarized in the wing?

7. The discussion states, that the loss of Ed leads to alterations in Ecad localization. Is the Ecad localization altered in RASSF8 and/or ed clones altered in the same way? Furthermore, the phenotypes of RASSF8 and ed (actomyosin cables that lead to exclusion of wildtype neighbors) do not fully overlap. A further discussion of the differences and the possible involvement of other factors/mechanism could be helpful.

8. Finally, the paper only has four Figures. We think it would be clearer for the reader if Figure S3 were included as one of the main Figures.

9. In the discussion, the authors state: “Interestingly, Ed depletion in the wing results in similar hexagonalisation and wing elongation defects as RASSF8 mutants”. The effect of Ed RNAi is quite a bit more subtle and the authors should acknowledge this.

Minor comments:
1. It would have been very helpful to introduce numbering of lines and pages.
2. The part of the introduction describing the wing development would benefit from the introduction of an overview scheme depicting developmental stages and wing regions/axes.
3. The introduction of the RASSF8 mutant would benefit from more details concerning the nature of the mutant, viability and fertility. Furthermore, the text is lacking an explanation why the RASSF8/Deficiency heterozygous tissues were used in Fig 1 and not for further experiments.
4. In the last part of the introduction the statement “Intriguingly, RASSF8 also has ASPP-independent functions, since RASSF8 mutant flies, unlike ASPP mutants, have a broad wing phenotype, indicative of abnormal PD axis extension.” refers to the present study without being mentioned so.
5. Second to last paragraph of the results. The authors introduce Echinoid as follows: “Ed is a large immunoglobulin (Ig) repeat trans-membrane homotypic adhesion molecule that cooperates with Ecad to mediate cell adhesion and sorting via the actomyosin network [63-66].” This is not really an accurate summary—while an essential role in cell adhesion was Takai’s original vision of the function of nectins, its now clear their role in mostly in cell sorting and defining boundaries between cell populations. I’d also note with regard to the 4th paragraph of the discussion that loss of Ed does not always affect Ecad localization—embryos develop to dorsal closure without it.
1. In Fig 1D and G the green tetragon line is covered up by the red octagon line 2. Fig S1F: The protein stained in red is not named within the figure/image.
3. Fig S3A: The clonal regions could be marked with -/-, +/+, +/-
4. Results “This suggests that N-terminal RASSF proteins have a general function in Par3/Baz recruitment.”: It seems that there is a “binding” missing

© 2021. Published by The Company of Biologists under the terms of the Creative Commons Attribution License (https://creativecommons.org/licenses/by/4.0/).

First revision

Author response to reviewers' comments

Dear Thomas,

I would like to submit a revised version of our manuscript entitled "**RASSF8- mediated transport of Echinoid via the exocyst promotes Drosophila wing elongation and epithelial ordering**". We are very grateful to the reviewers for their helpful comments, which have helped us improve the manuscript. Please see below for a detailed response to the reviewers' comments.

Best regards, Nic

We thank all three reviewers for their constructive comments. Here is our detailed response.

Reviewer 1 Comments for the Author:
1) page 8, "Interestingly, the localisation of Sec5, which is primarily cortical in the pupal wing, is not altered in RASSF8 mutant clones, suggesting that RASSF8 loss only affects vesicle-associated exocyst components or a subset of Sec15 exocyst complexes not associated with Sec5 (Figure S3B-B')." It seemed more likely to me that this would suggest that Sec5 is not sensitive to Sec15 loss, also in keeping with the subcomplex data from Macara.

This is indeed an interesting issue, although in fact *sec15* mutants do show mislocalisation of Sec5, at least in photoreceptor cells (see Figure 7 of Metha *et al* Neuron 2005), suggesting that the two subcomplexes are not fully independent in all contexts.

To clarify for the reader, we have changed the statement in the results to:
"Interestingly, the localisation of Sec5, which is primarily cortical in the pupal wing, is not altered in *RASSF8*
mutant clones, suggesting that RASSF8 is required for the localisation of a subset of exocyst components".

Then in the discussion we have added a sentence to put this result in the context of the exocyst sub-complexes: "Our data indicate that RASSF8 loss disrupts the localisation of Sec15 (subcomplex II), while Sec5 (subcomplex is not affected (Fig. 3, 4)."

2) It would be nice to have some quantitation of Ed and E-cad levels at the plasma membrane (levels actually look higher in the clonal region?). Or, alternatively, if the authors think the central phenotype is Ed breaks, could continuity be assayed (standard deviation of fluorescent intensities across an interface, for example)? This was the one part of the study that led to questions for me - given prior work and also given that many TM proteins would be expected to transit through REs, a little more clarity on the subject would be good. However, the Ed phenocopy of RASSF8 was nice. Not all data needs to fit perfectly, just was the one curious result in the paper for me.

As requested, we now include a quantification of E-cad levels in RASSF8 mutant clones and observe no effect (Figure S1F''').

The case of junctional Ed is interesting, but unfortunately very tricky to reliably address. As the reviewer will appreciate from Figure 5A-B, the Rab11-positive compartments that accumulate in *RASSF8*-mutant tissue are very closely apposed to the adherens junctions (both in X/Y and in Z). This is expected as the exocyst mediates the final steps of vesicle docking prior to fusion. We have looked at various methods to separate vesicular and junctional Ed in our stacks, for example subtracting or dividing the Ed and Rab11 channels to isolate junctional Ed for quantification (see an example below). Unfortunately, although we could in principle derive numbers from these analyses, we are not convinced that any of these methods don't result in loss of membrane Ed staining in the *RASSF8* mutant clones because of the close proximity of the stranded vesicles to the junctions and the inherent arbitrariness of thresholding the Rab11-positive and Rab11-negative areas. For this reason, we have not included the data in the manuscript.

© 2021. Published by The Company of Biologists under the terms of the Creative Commons Attribution License (https://creativecommons.org/licenses/by/4.0/).

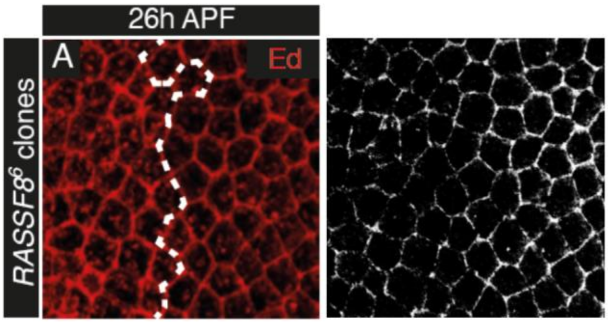

Reviewer Figure A: The original Figure 4A panel (left) and image obtained by dividing the Ed by the Rab11 channel (right).

3) A small item, has moderate Crumbs loss been looked at at these stages? It is another important TM protein that has been implicated in being trafficked through exocyst and Rab11 trafficking.

The reviewer is correct, *crumbs* mutant clones have junctional defects during pupal wing development (see Figure 3 in Salis, P et al Scientific Report 2017). Unlike *RASSF8* mutants, hexagonal packing is largely normal upon *crb* depletion, but the authors observed broken junctions and abnormal cell orientation in the plane of the tissue. As we had not observed a defect in Crb localisation in *RASSF8* mutants, we did not pursue this avenue.

Reviewer 2 Comments for the Author:
I have a few minor points:
1) Papers 1-4 are all old reviews. Perhaps the authors could update their reading list.

We have updated these references to newer reviews as requested.

2) Rab11 could mediate recycling or biosynthetic trafficking (as it does for example for E-cadherin in MDCK cells: Lock and Stow, MBC 2005). The authors should add evidence Ed recycling or biosynthetic transport or discuss these options appropriately.

It is true that the vesicles we observe could contain both newly synthesized and recycled Ed. As requested by the reviewer, we have amended the text to reflect this fact. In general, we have avoided the term “Recycling Endosomes” when referring to the enlarged Rab11 compartment and have instead used “compartments” or “vesicles”. We have also used “transport” or “trafficking” rather than ”recycling”, including in the title. In the discussion, we have added the following sentence:
“The exocyst and Rab11 are involved in both biosynthetic and recycling trafficking {Heider and Munson, 2012}, therefore RASSF8 could promote the delivery of newly synthesized and/or recycled Ed to the junctions.”

3) Hrs is not a lysosome marker. It is part of ESCRT-0 and a marker of ‘mature endosomes’ and detaches from the late endosomes before they reach the lysosome state.

We amended the text as requested.

4) Ed may also been a ‘homotypic’ adhesion molecules (which means it mediates adhesion between the same cell types) but the author presumably mean ‘homophilic’ adhesion molecule (meaning Ed on one cells binds to Ed on the adjacent cell). Ed is not a clear nectin homolog. Perhaps best to identify it as a nectin-like protein in term as AJ localization and binding to Afadin/Canoe.

We apologise for the mistake, we did mean homophilic, and have corrected this.

It is also true that Ed shares functional similarities with nectins rather than being a straightforward ortholog. We have therefore removed the word nectin from the title. As suggested, we only refer to Ed as “nectin-like”, and have added an explanatory sentence when Ed is first introduced in the results:
“Ed presents functional similarities to mammalian nectins: both are junctional components that belong to the Ig superfamily and recruit the F-actin binding protein Canoe (Cno; afadin in

© 2021. Published by The Company of Biologists under the terms of the Creative Commons Attribution License (https://creativecommons.org/licenses/by/4.0/).

mammals), but as their domain structure differs, Ed is considered nectin-like rather than being a nectin ortholog (Mandai, et al, 2013; Wei, et al 2005).”

Reviewer 3 Comments for the Author:
Major comments:
1. The central point of the paper concerns role of Rassf8 in localization/function of the exocyst and Rab11. They find striking effects. However, two issues detract from careful analysis of the data. Overall, the use of grey scales for single channel images would improve the presentation of the images—this is true in Figures 3 and 4 and in several of the Suppl. Figures. Second, they have images of sufficient quality that one could examine subcellular localization, but the magnifications used preclude this. They need to include closeups of images like those in Figure 3A-F—they do provide one example in Figure 4B. This was our most important issue with the data.

As suggested by the reviewer, we have made the following changes (note that figure panel numbering corresponds to the revised version of the ms, which is slightly different from the original version):
- Switching key panels to greyscale to improve visibility (Fig 3A, A’, A’’’, B, B’, B’’’, C, C’, C’’’, D, D’’, E, E’’, F, F’’;
  Fig. 4A, A’, B, B’, C, C’, D, D’; Fig. 5 A, A’, B, B’; Fig. S3A, A’, B, B’, C, C’; Fig. S4F, F’).
- Providing close-up images (Fig. 3A’’’, B’’’, C’’’, D’’, E’’, F’’).

On a more minor point, tell us in the main text whether the Sec15:GFP used was an insertion at the endogenous locus or an overexpressed protein, and give us a clearer indication of how +/+ tissues were distinguished from +/- tissues in Figure 3.

The Sec15::GFP construct is expressed under the control of the ubiquitin-63E promoter, which we frequently use to drive low-level ubiquitous transgene experiments. We now indicate this in the main text:
“We observed cytoplasmic accumulation of Sec15::GFP (expressed under the *ubiquitin-63E* promoter – see materials and methods) and Rab11 in RASSF8 clones at various time points (Fig. 3A-F”).”

We now indicate how the +/+ and +/- tissues are distinguished in the figure legend:
“In the merge channel, the genotypes of the clones are given (+/+ wild type – two copies of RFP, +/- heterozygous – one copy of RFP, -/- homozygous RASSF8 mutant – no copies of RFP).”

2. Concerning Figures 1B-G, 4G-L, S1A-K: As indicated in the text, these images and quantifications were taken at different positions in the wing, an overview image of the wing with a depiction of the actual position would be helpful. Also, a legend of the axes within wing images would be helpful.

We have added a diagram (Fig. 1A) that shows the positions at which the Ecad images for quantifying hexagonal packing are taken, as well as helping the reader navigate the stages we are examining in the manuscript (see minor point 2). The axes are shown on the diagram, and we consistently use the same orientation in the wing images throughout the manuscript, which is now indicated in the legend to Figure 1.

3. The text describing the results of the cell packaging could make clearer that the packaging represents the number of neighbors of a single cell.

We added the following sentence in the relevant part of the results:
“The polygon distribution indicates the number of neighbours of each individual cell, from tetragons (four neighbours) to octagons (eight neighbours).”

4. Results concerning Fig S3A/ Sec5 in RASSF8 clones: In the text it is stated, that the Sec5 amount in clones is not altered, however this is not really clear in the image. The conclusion would benefit from a quantification/line plot.

We now provide a line plot for this panel as suggested (Fig. 4B, B’) in the revised manuscript). Note that we have changed the image in this panel to a picture with a larger mutant clone than the

© 2021. Published by The Company of Biologists under the terms of the Creative Commons Attribution License (https://creativecommons.org/licenses/by/4.0/).

previous one for clarity.

The same is true for Figure S4F/ the statement, that there is no change in Ecad and Rab11 levels in baz clones.

We do not make any claims about Ecad localisation in *baz* clones.

As requested by the reviewer, we have added a quantification for Rab11 staining in *baz* clones (Fig. S4G). In agreement with the original submission, we observe a mild (1.1-fold), but statistically significant increase in Rab11 in *baz* mutant tissue compared to control. To allow the reader to appreciate the difference in Rab11 accumulation between *baz* and *RASSF8* mutants, we have normalised the data over the wild-type tissue in the same wings in both cases (see Fig. S4G and 5C). This confirms that Rab11 accumulation in the wing disc is much more pronounced in *RASSF8* (1.62-fold) than in *baz* clones (1.1-fold).

5. Figure S4A-E/ Holes in retina junctions: Is a similar disruption of junctions/ fragmentation of junctional markers detectable in wing tissues? And if not, is this due to a RASSF8-Sec15 independent function (as f. e. RASSF8-ASPP-Magi) that may not play a role in wing development?

Although we have not performed TEM on pupal wing disc, it is true that in the eye disc we observe breaks in Ecad staining (Langton et al, Curr Biol 2009), which is not the case in the pupal wing (this report). It is indeed possible that this is due to tissue specific function, as is the case for *ASPP*, which does not elicit a round wing phenotype like *RASSF8*.

6. It is stated several times, that the planar polarization/redistribution of junctional components is crucial for the probed tissue dynamics. Have they thought about the localization of planar polarized proteins in RASSF8 mutant tissues? And is Ed planar polarized in the wing?

In our mini-screen for membrane proteins that may be mislocalised in RASSF8 mutants, we looked at the core PCP member Flamingo (Fmi) and saw no effect. We have now added an image to the manuscript (Figure S4H- H'').

The suggestion that Ed might be planar polarised in the pupal is an interesting one, especially since Ed can be planar polarised at a single cell levels at boundaries of Ed expression, for example at clonal boundaries, where Ed is absent at the junctions of wild type cells that abut mutant cells (Wei et al Dev Cell 2005). Visually, however, Ed does not appear to be planar polarised in wild type wing tissue at the stages we examined. To further examine this issue, we took advantage of QuantifyPolarity, a planar polarisation quantification tool very recently published by the Strutt lab (Tan et al, Development 2021). We quantified Ed planar polarisation in wild type tissue, such as the control areas in our RASSF8 mosaics (see an example in Reviewer Figure B, left panel).
These data indicate that Ed is not planar polarised. To check that we properly applied the quantification procedure, we took the same images and artificially created an Ed expression gradient using Image J, and observed clear planar polarity using QuantifyPolarity (Reviewer Figure B, right panel). Therefore, Ed does not appear to be planar polarised in the pupal wing.

© 2021. Published by The Company of Biologists under the terms of the Creative Commons Attribution License (https://creativecommons.org/licenses/by/4.0/).

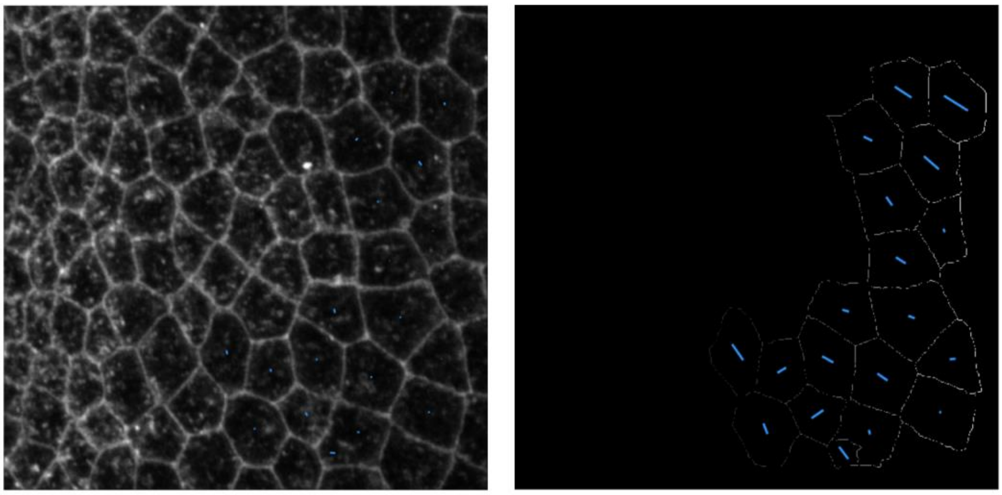

Reviewer Figure B: Confocal micrograph from a mosaic pupal wing containing RASSF8 mutant clones (left hand of the image). Note that the quantification of planar cell polarity has only been applied to the wild type area of the image (right hand of the image). The length and orientation of the blue bars shows the extent and orientation of planar polarity measured by QuantifyPolarity. Left panel: Ed is not planar polarised. Right panel: an artificial left-right Ed expression gradient has been applied to the image and shows that the quantification procedure works.

7. The discussion states, that the loss of Ed leads to alterations in Ecad localization. Is the Ecad localization altered in RASSF8 and/or ed clones altered in the same way? Furthermore, the phenotypes of RASSF8 and ed (actomyosin cables that lead to exclusion of wildtype neighbors) do not fully overlap. A further discussion of the differences and the possible involvement of other factors/mechanism could be helpful.

The changes in Ecad localisation and actomyosin contractility elicited by *ed* loss occur at clonal boundaries, and the reviewer is correct in that we do not observe these defects at *RASSF8* mutant clonal boundaries. It is not yet clear how Ed influences hexagonalisation in the wing, perhaps via an adhesion function, or via as yet unknown effector proteins. We have modified the fifth paragraph of the discussion to reflect this:

"How could disruptions in Ed trafficking lead to epithelial reordering defects? Like many Ig superfamily molecules, Ed can trans-dimerise (Islam et al., 2003; Rawlins et al., 2003a). Ed is also associated with the acto-myosin cytoskeleton via a direct interaction with the actin filament binding protein Canoe (Wei et al., 2005). So far, the majority of Ed functions have been related to cell sorting at Ed expression boundaries. Indeed, at the boundary of ed mutant clones, Ed is lost from the junctions of wild type cells that abut the mutant clones, inducing the assembly of a contractile acto-myosin cable that leads to apical constriction of the mutant cells (Wei et al., 2005). Acto-myosin contractility at the clone border, together with differential adhesion, leads to a cell sorting phenotype characterised by a smooth border between the mutant and wild type populations (Chang et al., 2011). Naturally occurring Ed expression boundaries can also trigger acto-myosin cable formation and drive cell sorting events in several morphogenetic processes, such as dorsal closure (Laplante and Nilson, 2006; Lin et al., 2007), ommatidial rotation (Fetting et al., 2009; Ho et al., 2010) and ovarian follicle cell segregation (Laplante and Nilson, 2006). However, as we did not observe any Ed expression boundaries in the pupal wing, and RASSF8 mutant clones do not display the characteristic round smooth border of ed mutant clones, Ed's role in hexagonalisation is likely to be distinct. Whether this role involves cytoskeletal modulation or an Ed adhesive function through homophilic association or heterophilic interactions with other partners such as its paralog Friend of Echinoid (Ozkan et al., 2013) remains to be investigated. Alternatively, as Ed has been shown to modulate several signalling pathways, such as Notch (Chandra et al., 2003; Escudero et al., 2003; Rawlins et al., 2003a), Hippo (Yue et al., 2012) and Epidermal growth factor receptor (Bai et al., 2001; Fetting et al., 2009; Islam et al., 2003; Rawlins et al., 2003b; Spencer and Cagan, 2003), it may be acting via cell-cell signalling."

© 2021. Published by The Company of Biologists under the terms of the Creative Commons Attribution License (https://creativecommons.org/licenses/by/4.0/).

8. Finally, the paper only has four Figures. We think it would be clearer for the reader if Figure S3 were included as one of the main Figures.

As suggested, we have moved most of the data from Figure S3 to a new main Figure 4.

9. In the discussion, the authors state: “Interestingly, Ed depletion in the wing results in similar hexagonalsation and wing elongation defects as RASSF8 mutants”. The effect of Ed RNAi is quite a bit more subtle and the authors should acknowledge this.

We have changed the sentence to:
“Ed depletion in the wing results in similar, **though less pronounced**, hexagonalisation and wing elongation defects to RASSF8 mutants…”

Minor comments:
1. It would have been very helpful to introduce numbering of lines and pages. We have done this.
2. The part of the introduction describing the wing development would benefit from the introduction of an overview scheme depicting developmental stages and wing regions/axes.

As suggested, we have made an overview scheme to help readers navigate the manuscript (Fig. 1A).

3. The introduction of the RASSF8 mutant would benefit from more details concerning the nature of the mutant, viability and fertility. Furthermore, the text is lacking an explanation why the RASSF8/Deficiency heterozygous tissues were used in Fig 1 and not for further experiments.

The *RASSF8^6* mutant is described in detail in Langton et al Curr Biol 2009, which we cite in the materials and methods. We now mention in the legend to Figure 1 that it is a null mutant. The cross to the deficiency is not strictly necessary, but useful to verify that the phenotype is similar between *RASSF8^6/RASSF8^6* homozygotes versus *RASSF8^6/Df* trans-heterozygotes. This test is the genetic definition of a null mutation. We now explain this in the legend to Figure 1.

4. In the last part of the introduction the statement “Intriguingly, RASSF8 also has ASPP-independent functions, since RASSF8 mutant flies, unlike ASPP mutants, have a broad wing phenotype, indicative of abnormal PD axis extension.” refers to the present study without being mentioned so.

We have corrected this:
“Intriguingly, RASSF8 also has ASPP-independent functions, since RASSF8 mutant flies, unlike ASPP mutants, have a broad wing phenotype, indicative of abnormal PD axis extension ((Langton et al., 2009) and this study).”

5. Second to last paragraph of the results. The authors introduce Echinoid as follows: “Ed is a large immunoglobulin (Ig) repeat trans-membrane homotypic adhesion molecule that cooperates with Ecad to mediate cell adhesion and sorting via the actomyosin network [63-66].” This is not really an accurate summary—while an essential role in cell adhesion was Takai’s original vision of the function of nectins, its now clear their role in mostly in cell sorting and defining boundaries between cell populations. I’d also note with regard to the 4th paragraph of the discussion that loss of Ed does not always affect Ecad localization—embryos develop to dorsal closure without it.

As suggested by the reviewer, we have deleted the reference to cell adhesion in this sentence in the results (“that cooperates with Ecad to mediate cell adhesion and sorting via the actomyosin network”). Following a comment from reviewer 2, we have also clarified that Ed has nectin-like properties, rather than being a true nectin ortholog: p11: “Ed presents functional similarities to mammalian nectins: both are junctional components that belong to the Ig superfamily and recruit the F-actin binding protein Canoe (afadin in mammals), but as their domain structure differs, Ed is considered nectin-like rather than being a nectin ortholog (Mandai et al., 2013; Wei et al., 2005).”

In response to this comment and main comment 7, we have also re-worked the relevant paragraph of the discussion to provide more context on the known roles of Ed in mediating cell sorting at

© 2021. Published by The Company of Biologists under the terms of the Creative Commons Attribution License (https://creativecommons.org/licenses/by/4.0/).

expression boundaries (see response to comment 7 above).

1.In Fig 1D and G the green tetragon line is covered up by the red octagon line.

We have made the red octagon line dashed in order to allow the reader to see the green tetragon line.

2.Fig S1F: The protein stained in red is not named within the figure/image.

We have added the name (RFP) to the relevant figure panel.

3.Fig S3A: The clonal regions could be marked with -/-, +/+, +/-

In Figure 3A, we had marked the clonal and heterozygous regions to clarify the fact that the het tissue already presents a Sec15 localisation defect. We have not done this in all other figures (including Figure S3A, which is now Figure 4A) as we believe this would be distracting to the reader.

4.Results “This suggests that N-terminal RASSF proteins have a general function in Par3/Baz recruitment.”: It seems that there is a “binding” missing

I am not sure where “binding” should be placed in this sentence. The sentence seems ok to me as it is.

Second decision letter

MS ID#: DEVELOP/2021/199731

MS TITLE: RASSF8-mediated transport of Echinoid via the exocyst promotes Drosophila wing elongation and epithelial ordering

AUTHORS: Eunice Ho Yee Chan, Yanxiang Zhou, Birgit L Aerne, Maxine V Holder, Anne Weston, David J Barry, Lucy Collinson, and Nicolas Tapon
ARTICLE TYPE: Research Article

I am happy to tell you that your manuscript has been accepted for publication in Development, pending our standard ethics checks.

Reviewer 1

Advance summary and potential significance to field

The authors have responded appropriately to reviewer comments. The revised manuscript is nicely done, and it should make a good addition to Development.
Congratulations on the nice study!

Comments for the author

The authors have responded appropriately to reviewer comments. The revised manuscript is nicely done, and it should make a good addition to Development.
Congratulations on the nice study!

© 2021. Published by The Company of Biologists under the terms of the Creative Commons Attribution License (https://creativecommons.org/licenses/by/4.0/).

Reviewer 3

Advance summary and potential significance to field

Promoting tissue morphogenesis without compromising tissue integrity depends on dynamic reorganization of adherens junctions. Here the authors describe a novel function of the RA-domain protein RASSF8 as a player in AJ component exocytosis and overall tissue dynamics. Building on earlier work on the function of the RASSF8-ASPP complex in the tissue specific regulation of junctional components like Magi and Bazooka, the authors provide convincing evidence for an ASSP-independent role of RASSF8.

They show that RASSF8 acts as an anchor for recycling endosomes that deliver actomyosin regulating factors to the junctions. They make use of the developing Drosophila wing to study tissue and junction dynamics in vivo by combining qualitative and quantitative approaches. Furthermore, they combine their interesting findings with biochemical protein-interaction approaches. The study provides further insights into these processes with are crucial for development but about which questions remain. The data are well presented and well explained and will be interesting for a broad readership interested in both tissue and junction dynamics and also intracellular trafficking. We had some relatively minor suggestions and the authors have effectively addressed them--very nice work!

Comments for the author

Lovely work!

© 2021. Published by The Company of Biologists under the terms of the Creative Commons Attribution License (https://creativecommons.org/licenses/by/4.0/).
